# Review of the mechanism of infection induced cerebral small vessel disease

**DOI:** 10.3389/fimmu.2025.1594891

**Published:** 2025-05-26

**Authors:** Jiamei Song, Yiqin Wang, Zhaoxia Xie, Jiayi Wei, Jue Wang

**Affiliations:** ^1^ Department of Neurology, Shengjing Hospital of China Medical University, Shenyang, China; ^2^ Department of Developmental Cell Biology, Key Laboratory of Cell Biology, Ministry of Public Health, and Key Laboratory of Medical Cell Biology, Ministry of Education, China Medical University, Shenyang, China

**Keywords:** cerebral small vessel disease, pathogen infection, neuroinflammation, pathogenesis mechanisms, blood-brain barrier disruption

## Abstract

Cerebral small vessel disease (CSVD) refers to a group of pathological syndromes that affect the brain’s microcirculation. These conditions involve damage to small arteries, arterioles, capillaries, venules, and small veins. Cerebrovascular risk factors, immunosenescence, and inflammatory responses contribute to the pathogenesis of cerebral small vessel disease. The global impact of Severe Acute Respiratory Syndrome Coronavirus 2 (SARS-CoV-2) has drawn significant attention to chronic inflammation caused by infections. Research into the mechanisms by which infections induce CSVD has made continual advancements. It is imperative to reassess the importance of managing infections and the chronic inflammatory phase that follows, highlighting their critical role in the pathogenesis. Our focus encompasses SARS-CoV-2, Human Immunodeficiency Virus (HIV), Hepatitis C Virus (HCV), Zika Virus(ZIKV), Treponema pallidum, as well as the microbial communities within the gut and oral cavity. These pathogen infections and chronic inflammation can contribute to CSVD through mechanisms such as neuroinflammation, blood-brain barrier disruption, microthrombosis, and endothelial cell damage, thereby promoting the occurrence and progression of the disease. This highlights the need for detailed mechanistic research on CSVD associated with these pathogens. Furthermore, we hope that in the future, we will be able to devise targeted prevention and treatment strategies for CSVD based on the unique characteristics of the pathogenic mechanisms associated with various infections.

## 1 Introduction

Cerebral small vessel disease (CSVD) is a collective term that encompasses a spectrum of disorders affecting the small blood vessels in the brain. It is a leading cause of pathological processes, including stroke, dementia, and aging, across the globe (1). Moreover, the primary clinical manifestations of CSVD extend to psychiatric disorders, personality changes, compromised balance, abnormal gait, and urinary incontinence, positioning it as one of the significant contributors to the immense health burden on the global population (2, 3). The imaging manifestations of CSVD include small subcortical infarcts, lacunes, white matter (WM) hyperintensities (WMHs), enlarged perivascular spaces, microhemorrhages, and brain atrophy (4). Age is the most significant risk factor for cerebral small vessel disease, with approximately 5% of individuals over the age of 50 being affected, and the prevalence approaches nearly 100% in those over 90 years old (5). Additional risks include male sex, smoking, hypertension, and diabetes (6, 7). The pathogenesis remains poorly understood, and no effective treatments exist. Incomplete mechanistic insights hinder prevention and treatment. Current therapeutic approaches focus on strict blood pressure control, antiplatelet therapy, statins, and thrombolytic treatment. There is an urgent need for further exploration of the pathogenesis to refine treatment strategies (5).

Previous studies have categorized cerebral small vessel disease as a subtype of ischemic stroke driven by pathological cascades triggered by microthrombi occluding small blood vessels. However, emerging evidence suggests inflammation and endothelial dysfunction may represent core mechanisms of CSVD pathogenesis. Neuroinflammation’s role in CSVD has become a research focus. Typically, neuroinflammation is characterized as a complex defensive response mounted by the central nervous system against microbial infections, traumatic brain injury, or the clearance of other toxic substances (8), the condition is categorized into infectious and non-infectious types, with infection serving as the primary instigator of inflammation. Chronic inflammatory states promote the adhesion of leukocytes to the vascular endothelium, thereby compromising endothelial function. Inflammatory cells converge around the blood vessels, causing degradation of the blood-brain barrier and widening of the perivascular space. The release of inflammatory mediators activates microglial cells, amplifying the inflammatory cascade, which can culminate in white matter lesions and potentially induce structural changes in the vasculature, such as arteriosclerosis and disrupted autoregulation (9). Microglia in the central nervous system share functional similarities with macrophages in other tissues. Under healthy conditions, microglia remain quiescent; during infection or inflammation, they become activated professional phagocytes. This activation resolves infections but may also disrupt tissue homeostasis (10). In infections, astrocytes aid pathogen clearance via antimicrobial responses, yet prolonged inflammation damages neural tissue (11).

**Figure 1:**
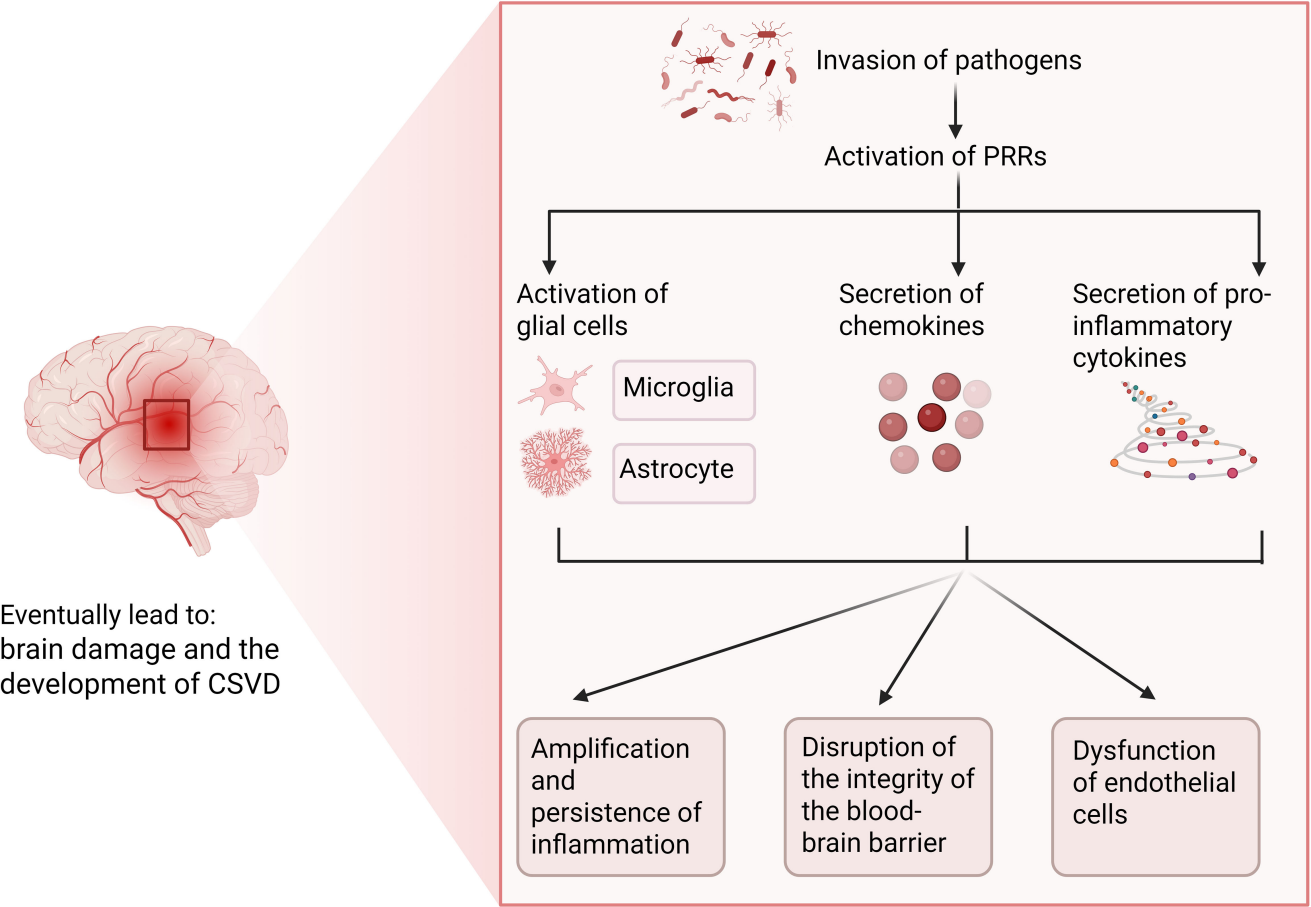
Potential role of infection-induced neuroinflammation in CSVD. Figure created with BioRender.com

Neuroinflammation precipitates not merely the activation of glial cells within the central nervous system, but also facilitates the secretion of pro-inflammatory cytokines and chemokines, ultimately precipitating the migration of peripheral immune cells. Moreover, this process may induce modifications in the integrity of the blood-brain barrier (BBB), resulting in enhanced permeability and its subsequent compromise (12). BBB breakdown drives cognitive impairment in CSVD. The BBB is primarily formed by capillary endothelial cells, which are tightly connected and restrict the entry of pathogens into the central nervous system (CNS). Astrocytes establish connections with the capillary endothelial cells through their perivascular endfeet and play a crucial role in maintaining the integrity of the BBB (13). Inflammation triggered by pathogens usually originates at the endothelial cells of the blood-brain barrier, which are equipped with molecular mechanisms for sensing bacterial and viral antigens (14). In response to microbial invasion, pattern recognition receptors (PRRs) activate innate immune cascades by initiating phagocytosis and pathogen clearance. Toll-like receptors (TLRs), expressed by glia and neurons, are critical PRR subtypes. During infection, pathogen- or endogenous-derived signals activate PRRs, triggering adaptive immunity, astrocyte activation, and chemokine release. Reactive astrocytes secrete VEGF, ROS, and glutamate, causing neurodegeneration, BBB hyperpermeability, and amplified local inflammation (11). BBB disruption during infection may be transient (15). Persistent inflammation, however, can cause chronic BBB degradation, leading to severe brain injury and CSVD symptoms (**Figure 1**).

**Figure 2:**
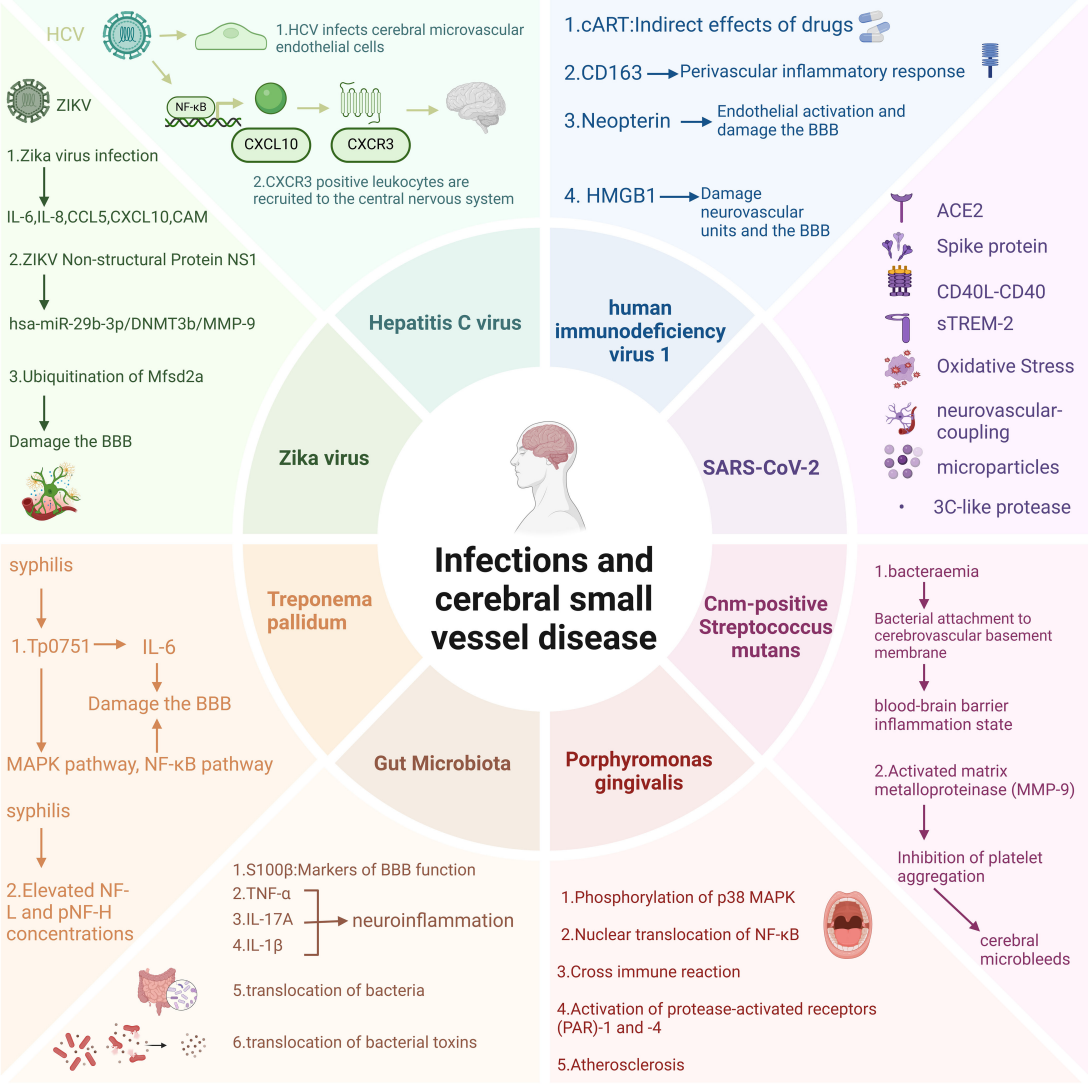
Potential impact mechanisms of pathogen infection on CSVD. Figure created with BioRender.com.

Current investigative endeavors may predominantly concentrate on the correlation between non-infectious neuroinflammation and cerebral small vessel disease. Evidence suggests that the aging process within the central nervous system is linked to chronic sterile low-grade inflammation, which predisposes to the accelerated deterioration of CSVD. Consequently, the bulk of research is centered on anti-inflammatory therapeutic strategies aimed at forestalling the induction and exacerbation of CSVD by overly exuberant neuroinflammatory activity (16). Reports on scenarios involving pathogen invasion are relatively scarce; however, it has come to our attention that there are accounts suggesting a communication and coordination between the nervous system and the immune system during the processes of pathogen invasion, inflammatory states, and immune dysregulation (17). This manuscript chiefly summarizes the influence of inflammation induced by multiple pathogen infections on cerebral small vessel disease (**Figure 2**, **Table 1**). This study aims to advance the research on the pathogenic mechanisms of CSVD in the context of infection and to offer novel insights for the prevention and treatment of cerebral small vessel disease.

**Table 1:** Possible mechanisms of the effects of a variety of pathogens on CSVD.

Type of Pathogen, Authors & Year of Publication	Study Design	Research Subjects	Sample Size(n)	Research indicators	Results	Potential mechanisms affecting CSVD	Limitations
SARS-CoV-2, Laura Pellegrini et al., 2020 (18)	Experimental Study	Human brain organoids	NR	Viral Load, expression of specific proteins post-infection (such as ACE2 and TMPRSS2), viral replication capacity	Demonstrated ACE2 expression in mature choroid plexus cells and infection of these cells by SARS-CoV-2	The direct invasion, compromise of the BBB	The study is limited to organoids, which may not fully represent human brain conditions;
SARS-CoV-2, Rongrong Chen et al., 2021 (19)	Bioinformatics Analysis	Human and Mouse Brains	Humans (n=55); Mice (n=11)	ACE2 Receptor Expression	The brain infection by SARS-CoV-2 may elicit symptoms within the central nervous system	The direct invasion	A deficiency in additional research precludes confirmation of its relationship with cerebrovascular disease
SARS-CoV-2, Yeshun Wu et al., 2020 (20)	Review	Patients with COVID-19	NR	Neurological involvement post-infection	COVID-19 infection can affect the nervous system	The Spike protein of SARS-CoV-2 interacts with ACE2 in capillary endothelial cells; concurrently, the virus has the potential to disrupt the BBB and target the vascular system	Potential biases in case reporting
SARS-CoV-2, Aldo Bonaventura et al., 2021 (21)	Review	COVID-19 patients	NR	Endothelial dysfunction, immunothrombosis	Describes the role of endothelial dysfunction and immunothrombosis in the pathogenesis of COVID-19	Endothelial dysfunction and immunothrombosis may have cerebrovascular effects	The study is a review and may not include original data; potential biases in literature review
SARS-CoV-2, Hassan M Otifi et al., 2022 (22)	Review	Covid-19 patients	A total of 348 COVID-19 patients were mentioned	Not specified	Describes the role of endothelial dysfunction in the pathogenesis of COVID-19, including direct viral-induced endothelial injury, uncontrolled immune & inflammatory response, imbalanced coagulation homeostasis	Endothelial dysfunction	Potential biases in data interpretation
SARS-CoV-2, Brandon J DeOre et al., 2021 (23)	Experimental study using a 3D-BBB microfluidic model	Blood-brain barrier model	NR	Disruption of BBB integrity by SARS-CoV-2 spike protein	Demonstrates that SARS-CoV-2 spike protein disrupts BBB function, potentially involving RhoA activation	Disruption of the BBB	Potential biases in data interpretation
SARS-CoV-2, Elizabeth M Rhea et al., 2021 (24)	Experimental study	Mice	A total of 204 mice were mentioned	Not specified	SARS-CoV-2 S1 protein traversal across the murine blood-brain barrier	Neuroinvasion and inflammation may contribute to cerebrovascular damage	Limited to murine model
SARS-CoV-2, Luca Perico et al., 2024 (25)	Review	Not specified	NR	SARS-CoV-2 spike protein interaction with endothelial cells	The effects of SARS-CoV-2 spike protein on endothelial cells were summarized	Immune system hyperactivation and inflammation may lead to endothelial dysfunction	Review-based, lacks original experimental data
SARS-CoV-2, Susana Boluda et al., 2023 (26)	Experimental study	COVID-19 patients	3 biopsies and 3 autopsies	SARS-CoV-2 spike protein localization in the Golgi apparatus of brain endothelial cells and interaction with furin	The association between SP and furin has implications for the pathogenesis of COVID-associated microangiopathy	The interaction between SARS-CoV-2 spike protein and furin may contribute to cerebral microangiopathy, affecting CSVD	Small sample size
SARS-CoV-2, Fabricia L Fontes-Dantas et al., 2023 (27)	Experimental Study	Mice	86 patients	SARS-CoV-2 spike protein and TLR4-mediated cognitive dysfunction	TLR4 serves as a pivotal target for the prolonged cognitive impairment following COVID-19	Spike protein may contribute to cognitive dysfunction through TLR4-mediated pathways, potentially affecting CSVD	Lack of longitudinal evaluation
SARS-CoV-2, Nallely Garcia-Larragoiti et al., 2023 (28)	Observational Study	Recovered COVID-19 Patients	68 patients and 23 healthy controls	Inflammatory and prothrombotic biomarkers	Persistence of sequelae in recovered COVID-19 patients	Inflammatory and prothrombotic biomarkers may contribute to chronic vascular changes, potentially affecting CSVD	Lack of further study
SARS-CoV-2, Hind Hamzeh-Cognasse et al., 2021 (29)	Observational study	Early-stage SARS-CoV-2 infected patients	29 patients and 26 convalescent patients	Platelet-derived sCD40L	Elevated levels of platelet-derived sCD40L in early-stage SARS-CoV-2 infection	Inflammation and platelet abnormalities	Limited number of cases
SARS-CoV-2, Yongjian Wu et al., 2021 (30)	Experimental study	SARS-CoV-2 infected patients Mice	103 patients and 50 healthy controls	TREM-2	TREM-2 identified as a modulator in T cells during SARS-CoV-2 infection	Inflammatory response and tissue damage	Nil
SARS-CoV-2, Gilead Ebiegberi Forcados et al., 2021 (31)	Review	SARS-CoV-2 infected individuals	NR	Oxidative stress and inflammation	Oxidative stress and inflammation are associated with the progression of COVID-19 and the response to therapeutic interventions	The oxidative stress induced by SARS-CoV-2 may potentially lead to CSVD	Nil
SARS-CoV-2, Mikhail A Hameedi et al., 2022 (32)	Experimental study	SARS-CoV-2 3C-like protease (3CLpro)	NR	Cleavage of NEMO by 3CLpro	SARS-CoV-2 3CLpro cleaves NEMO,deregulating the host immune response	Immune disorders, chronic inflammation	Lack of further study
SARS-CoV-2, Jan Wenzel et al., 2021 (33)	Experimental study	SARS-CoV-2-infected brain endothelial cells	17 patients and 23 healthy controls	Cleavage of NEMO by SARS-CoV-2 Mpro	Microvascular brain pathology in COVID-19 patients	Lesion of the microvasculature.	Nil
SARS-CoV-2, Cameron D Owens et al., 2024 (34)	Review	COVID-19 patients	NR	Impaired neurovascular coupling	Cognitive deficits in COVID-19 patients	Neurovascular coupling impairment as a mechanism	Nil
SARS-CoV-2, Che Mohd Nasril Che Mohd Nassir et al., 2021 (35)	Review	COVID-19 patients	NR	MPs	MPs may be implicated in the pathogenesis of CSVD	Microparticles as microthrombogenic risk factors	Lack of further study
HIV, Virawudh Soontornniyomkij et al., 2014 (36)	Prospective cohort study	HIV patients on protease inhibitor therapy	144 autopsy cases of HIV infection	Protease inhibitor exposure	Increased risk of cerebral small vessel disease	Direct toxicity, metabolic abnormalities	Limited sample size
HIV, Richard W Price et al., 2013 (37)	Cross-sectional study	HIV-1-positive individuals	20 neurologically asymptomatic (NA) subjects, 12 HAD patients, and 19 patients receiving suppressive therapy, 20 healthy controls.	Neopterin and other indicators	Neopterin primarily reflects the stimulation of interferon-γ and its levels increase concomitantly with the progression of systemic HIV infection.	Inflammation and immune response	Lack of longitudinal data
HIV, Tricia H Burdo et al., 2011 (38)	Observational study	HIV-infected individuals (early and chronic phases)	30 chronic HIV infected individuals, 14 early HIV infected individuals, and 29 HIV negative individuals	Soluble CD163 levels	Elevated soluble CD163 associated with HIV activity	Immune response	Lack of longitudinal data
HIV, Marius Trøseid et al., 2010 (39)	Prospective cohort study	HIV-1 infected individuals	32 HIV-1-positive patients who had responded to antiretroviral therapy with undetectable viremia after 2 years, 10 nonresponders and 19 healthy controls	Plasma levels of LPS and HMGB1	Elevated levels of LPS and HMGB1 associated with high viral load;	Immune response	Nil
HCV, Nicola F Fletcher et al., 2012 (40)	Experimental study	Hepatitis C infected individuals	10 patients with HCV infection and 3 patients without HCV infection	HCV RNA levels in brain tissue, expression of viral entry receptors	HCV RNA detected in brain tissue; endothelial cells support viral entry and replication	Apoptosis of brain microvascular endothelial cells and increased endothelial permeability	Nil
HCV, Lydia Yarlott et al., 2017 (41)	Review	HCV-infected patients	NR	Neurological and psychiatric disorders	Detailed review of the relationship between HCV infection and neurological/psychiatric disorders	HCV-induced neuroinflammation, blood-brain barrier disruption	Lack of original research data
HCV, Yuan Liu et al., 2016 (42)	Experimental study	Human brain microvascular endothelial cells	NR	Hepatitis C virus infection, CXCL10 elevation	HCV infection induced CXCL10 elevation in brain microvascular endothelial cells	CXCL10 elevation may contribute to vascular inflammation and CSVD	Nil
Zika virus, Nilda Vanesa Ayala-Nunez et al., 2019 (43)	Experimental study	Monocytes, neural cells	NR	Zika virus infection	Enhanced monocyte adhesion and transmigration, facilitating viral entry into the CNS	May contribute to neuroinflammation and vascular damage	Nil
Zika virus,Utkarsh Bhardwaj et al., 2023 (44)	Experimental study	Human brain microvascular endothelial cells	NR	Zika virus NS1, hsa-miR-29b-3p, DNMT3b, MMP-9 pathway	ZIKV NS1 suppressed VE-cadherin, potentially affecting BBB integrity	May contribute to BBB disruption and subsequent CSVD	Nil
Zika virus,Jia Zhou et al., 2019 (45)	Experimental study	Brain microvascular endothelial cells	NR	Zika virus, Mfsd2a degradation	ZIKV degraded Mfsd2a, disrupting lipid homeostasis in hBMECs	Zika virus infection may disrupt the neurovascular microenvironment	Lack of further study
Syphilis, Simin Lu et al., 2022 (46)	Experimental study	bEnd3 cells	NR	Treponema pallidum Tp0751	Tp0751 promotes apoptosis and IL-6 secretion in bEnd3 cells, altering tight junction protein expression	May contribute to blood-brain barrier disruption	*In vitro* study; may not fully reflect *in vivo* conditions
Syphilis, Dong-Mei Xu et al., 2020 (47)	Prospective study	92 non-HIV-infected syphilis	23 patients with symptomatic neurosyphilis, 51 patients with asymptomatic neurosyphilis, and 18 patients with latent syphilis as control group	Neurofilament light subunit (NF-L) and phosphorylated neurofilament heavy subunit (pNF-H) in CSF	Elevated levels of NF-L and pNF-H in symptomatic neurosyphilis patients.	NF-L and pNF-H are associated with CSVD	Relatively small, age-mismatched sample size
Gut Microbiota, Bernard Fongang et al., 2023 (48)	Observational study	Participants in the Framingham Heart Study	972 participants	Gut Barnesiella intestinihominis abundance	Decreased abundance of gut B. intestinihominis associated with higher CSVD burden	Altered gut microbiota may influence CSVD	Potential confounding factors
Gut Microbiota, Yachen Shi et al., 2023 (49)	Observational study	Patients with cerebral small vessel disease	64 CSVD patients and 18 matched healthy controls	Gut microbiota composition	Altered gut microbiota composition associated with CSVD	Dysbiosis may contribute to CSVD pathogenesis	Lack of animal studies to validate the findings
Gut Microbiota, Wei Cai et al., 2021 (50)	Observational study	Patients with arteriosclerotic CSVD	55 arteriosclerotic CSVD patients and 62 healthy controls	Gut microbiota composition	Gut microbiota induces higher IL-17A production in neutrophils	State of inflammation	Lack of further study
Gut Microbiota, Sheng Liu et al., 2023 (51)	Observational study	CADASIL patients	24 CADASIL patients and 28 healthy controls	Gut microbiota composition	Gut microbes exacerbate systemic inflammation and behavior disorders	Microbiota-induced inflammation may contribute to CADASIL pathogenesis	The sample size was relatively small
Oral Microbiota, Clemens Walter et al., 2004 (52)	Experimental study	Human umbilical vein endothelial cells	NR	Porphyromonas gingivalis strains ATCC 53977 and DSMZ 20709	Both strains adhered to and infected endothelial cells, triggering signal transduction pathways and increased expression of endothelial adhesion molecules	Endothelial cell activation may contribute to vascular inflammation	Lack of further study
Oral Microbiota, Ghazal Aarabi et al., 2018 (53)	Review	patients with chronic oral infections (gingivitis/periodontitis)	NR	Association with CSVD	Oral infections may be associated with CSVD	Inflammation and systemic effects of oral infections may contribute to CSVD	Limited by the observational nature of the review
Oral Microbiota, A Lourbakos et al., 2001 (54)	Experimental study	Human platelets	NR	Gingipains from P. gingivalis	Gingipains induce platelet aggregation	Gingipains may contribute to thrombosis	Nil
Oral Microbiota, Satoshi Hosoki et al., 2020 (55)	Observational study	Patients with cerebral microbleeds	A total of 111 patients were identified; 21 (19%) with *cnm*-positive *S.mutans* and 90 (81%) without.	Streptococcus mutans with cnm gene	Oral carriage of S. mutans with cnm gene associated with increased CMBs	Inflammatory response and destruction of the BBB	Potential risk of selection bias

COVID-19, Coronavirus disease 2019; ACE2, angiotensin-converting enzyme 2; BBB, blood-brain barrier; RhoA, Ras homolog gene family member A; TLR4,toll-like receptor 4; sCD40L, soluble CD40 ligand; TREM-2, triggering receptor expressed on myeloid cells-2; NEMO, the nuclear factor (NF)-κB essential modulator; 3CLpro, 3C-like proteinase; SARS-CoV-2 Mpro, the SARS-CoV-2 main protease; MPs, Circulating Microparticles; CD163, The hemoglobin (Hb) scavenger receptor; LPS, lipopolysaccharide; HMGB1, High mobility group box 1; CXCL10, C-X-C motif Chemokine Ligand 10; NS1, the non-structural protein NS1 of ZIKV; Mfsd2a, a sodium-dependent lysophosphatidylcholine symporter; Tp0751, The T. pallidum adhesin Tp0751; CADASIL, Cerebral autosomal dominant arteriopathy with subcortical infarcts and leukoencephalopathy.

NR stands for “Not Reported”.

## 2 SARS-CoV-2 and CSVD

**Figure 3:**
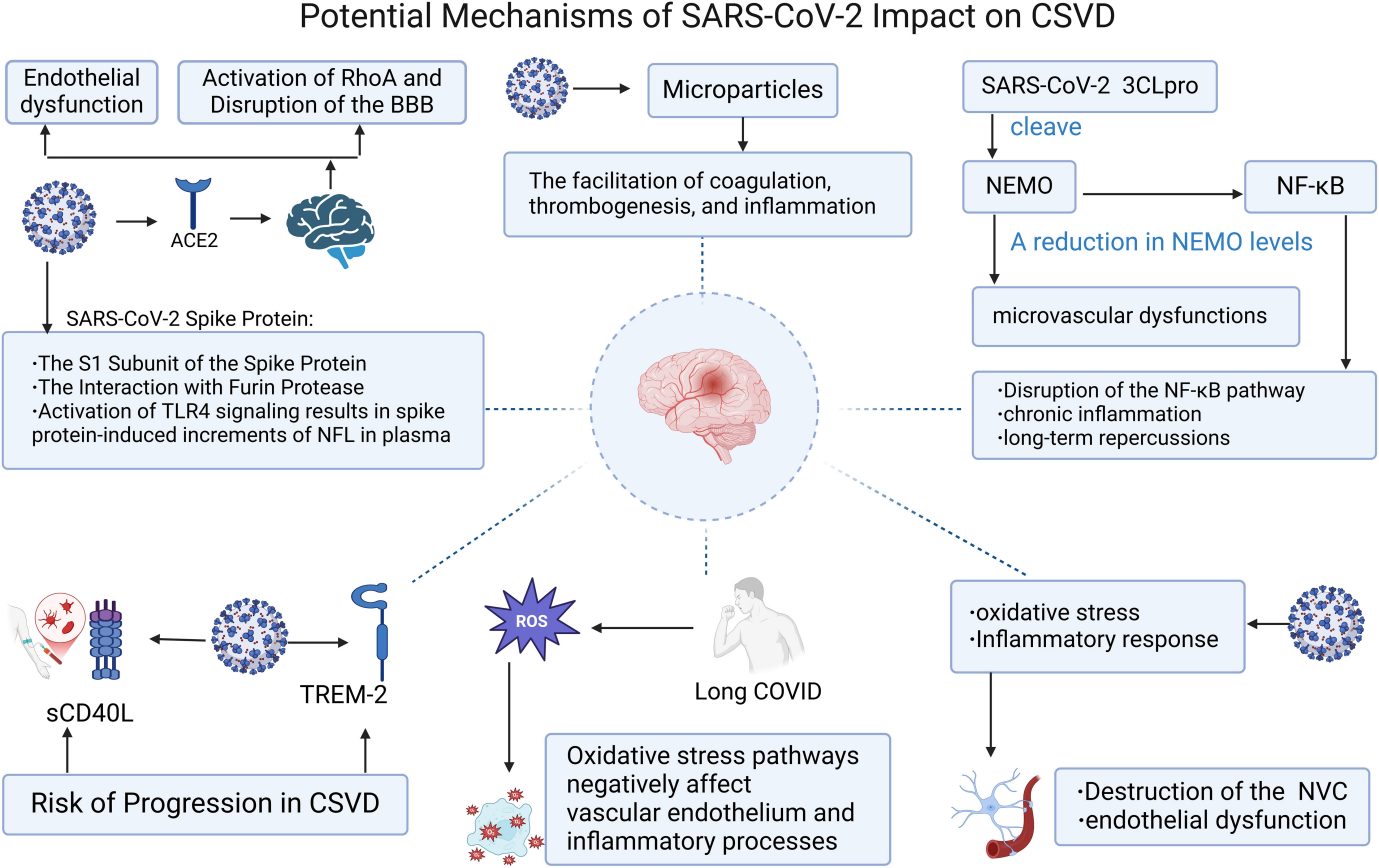
Potential mechanisms of SARS-Cov-2 impact on CSVD. Figure created with BioRender.com.

By the end of 2019, the appearance of a novel coronavirus named SARS-CoV-2 led to the outbreak of a rare form of viral pneumonia also termed COVID-19 (56). Coronaviruses belong to the family Coronaviridae within the order Nidovirales. Named for the crown-like spikes on their surface, coronaviruses measure 65–125 nanometers in diameter and possess a single-stranded RNA as their nucleic material (57). The virus exhibits a high degree of transmissibility, predominantly spreading through respiratory droplets and other modes, and has disseminated across the globe at an unprecedented rate (58). The initial symptoms observed in patients with COVID-19 encompass cough, mild fever, shortness of breath, nausea, and diarrhea, among others. Critically ill patients may experience severe complications such as pulmonary embolism, stroke, and myocardial infarction, which can involve multiple organ systems (59). In the realm of the nervous system, the most commonly encountered manifestations include alterations in taste and smell, headache, changes in level of consciousness, cognitive impairment, and neuropsychiatric symptoms. Less frequent presentations encompass transverse myelitis, seizure activity, rhabdomyolysis, cranial nerve palsies, and Guillain-Barré syndrome (60). Coronavirus genomic architecture enables novel mutations via cross-species recombination in diverse hosts, leading to sustained human health impacts, particularly severe neurological consequences that demand urgent attention (61). Elderly COVID-19 patients exhibit elevated risks of cerebrovascular complications and cognitive decline, potentially progressing to cerebral small vessel disease (CSVD). Despite unresolved etiology, systemic SARS-CoV-2 effects, inflammatory thrombosis, and elevated ROS levels collectively drive vascular dysfunction through synergistic pathogenic mechanisms. Moreover, the small vessel lesions in patients infected with COVID-19 are commonly observed in the subcortical white matter, corpus callosum, periventricular white matter, and subcortical regions. The pathological manifestations of this CSVD differ from those caused by other known etiologies, such as acute respiratory distress syndrome (ARDS) and hypertension, leading to a reasonable hypothesis that SARS-CoV-2 may induce CSVD through unique pathological mechanisms (62) (**Figure 2**). There is emerging evidence from certain research outcomes that may substantiate the phenomenon of SARS-CoV-2 gaining entry into the brain, including the virus’ potential to be internalized via endocytosis at neuronal terminals for retrograde transport, to cross the blood-brain barrier to infiltrate the brain, and to penetrate the brain through the infiltration of infected immune cells (63). These potential implications for cerebrovascular function warrant attention. Consequently, investigating the long-term neurological sequelae of COVID-19 and delineating the interplay between inflammation, endothelial injury, and cerebral small vessel disease is of paramount importance. As shown in **Figure 3**.

### 2.1 COVID-19 may affect CSVD through angiotensin-converting enzyme 2 and spike protein

The spike glycoprotein S of SARS-CoV-2 exhibits the capability to interact with the angiotensin-converting enzyme 2 of the host, thereby mediating the amalgamation of the viral envelope with the cellular membrane of the target cell. This interaction ultimately leads to the discharge of viral RNA into the cytoplasm of the host cell, subsequently triggering a cascade of detrimental impacts on the human physiological system (64). Low levels of ACE2 expression have been detected within cerebral endothelial cells, the choroid plexus, and the ventral posterior nucleus of the thalamus (18, 19). Additionally, the presence of SARS-CoV-2 has been detected in cerebrospinal fluid via gene sequencing, indicating a potential for direct invasion and infection of the nervous system through the ACE2 receptor. Furthermore, it suggests that the virus may transform into a persistent infection within the nervous system, possibly mediated by the combined effects of immune mechanisms (20). The SARS-CoV-2 virus, by virtue of its spike protein binding to the ACE2 receptor on endothelial cells, can precipitate endothelial dysfunction, dysregulation of coagulation homeostasis, and the formation of microvascular immunothrombosis. This process may also be accompanied by complement activation, increased endothelial barrier permeability, and compromised vasodilatory capacity (21, 22). Given that ACE2 is expressed within the cerebral vasculature, the binding of the virus to this receptor may activate RhoA, a key molecule regulating the cytoskeleton and tight junctions of endothelial cells, potentially leading to the disruption of the blood-brain barrier (23). This constellation of phenomena may underpin the pathogenesis of COVID-19-induced cerebral small vessel disease.

Upon the binding of the coronavirus spike protein to ACE2, the protein is enzymatically dissected by proteases within the host cell into two distinct subunits, S1 and S2. The S1 subunit is posited as a pivotal causative agent in the induction of endothelial dysfunction, with the potential for the cleaved S1 segment to permeate the blood-brain barrier (24). *In vitro* experiments show that the spike protein disrupts blood-brain barrier integrity, triggering endothelial pro-inflammatory responses and upregulating matrix metalloproteinases. This disruption reduces junctional proteins and increases brain microvascular permeability. Intravenous S1 protein injection in mice causes S1 accumulation in brain endothelial cells, correlating with endothelial injury and elevated C5b-9 levels. (25). The spike protein has the capacity to be transported in the bloodstream, cross the blood-brain barrier, and ultimately enter endothelial cells via endocytosis. The spike protein can bind to a serine protease known as Furin and accumulate in the Golgi apparatus. Furin is implicated in the metabolism of the SARS-CoV-2 spike protein and is highly expressed in vascular endothelial cells, where it can regulate endothelial permeability. The intimate association and interaction between the spike protein and Furin may be related to the pathogenesis of microvascular diseases associated with COVID-19 (26). COVID-19-induced brain dysfunction correlates with TLR4 signaling in microglia driven by spike proteins. TLR4 activation elevates plasma NFL levels via spike proteins, driving delayed neuroinflammation and cognitive dysfunction. These findings mirror cerebral small vessel disease pathology and clinical features (27). Although it is currently not fully clear whether CSVD can be linked to the COVID-19 spike protein, Furin protease, and other factors, future research may focus on patients with prolonged COVID-19 infection who exhibit cognitive impairment or other neurological symptoms. Efforts should be made to investigate the potential connections through further basic experiments, clinical symptom assessments, and imaging studies.

### 2.2 SARS-CoV-2 influences CSVD by enhancing the secretion of inflammatory factors

Infection with SARS-CoV-2 can lead to the upregulation of certain pro-inflammatory cytokines and chemokines, potentially resulting in the onset of cytokine release syndrome (CRS). CRS is characterized by excessive activation of immune cells and elevated levels of circulating cytokines, representing a systemic inflammatory state that may exacerbate disease severity and pose a life-threatening risk (65). It has been reported that inflammatory biomarkers such as interleukin-6 (IL-6) and C-reactive protein (CRP) are closely associated with the severity of the novel coronavirus infection (66–70). These biomarkers are also intricately linked to the pathogenesis of cerebral small vessel disease, and in comparison to individual biomarkers, the combined assessment of multiple biomarkers associated with CSVD may offer a more comprehensive explanation of the underlying pathological processes (71, 72). SARS-CoV-2 infection may cause long COVID (LC), a prolonged condition marked by pathological persistence beyond 12 weeks post-acute infection. LC involves sustained dysfunction across respiratory, neurological, cardiovascular, metabolic, and psychosocial systems. Elevated IL-6, D-dimer, PAI-1, and sCD40L levels confirm chronic inflammation and active immunothrombosis in LC patients. These abnormalities drive endothelial dysfunction and coagulation dysregulation. (28, 73), the prolonged persistence of such aberrant states may predispose to the development of cerebral small vessel disease.

Upon reviewing the literature, we postulate that tumor necrosis factor (TNF) and its receptors, CD40L/CD40, along with the cell surface receptor sTREM-2, are implicated in COVID-19-induced CSVD. Firstly, CD40, a member of the TNF receptor superfamily (TNFRSF), and CD40L, a member of the TNF superfamily (TNFSF), both participate in a multitude of immunological reactions within the body. CD40 is predominantly constitutively expressed in B cells and myeloid cells, whereas CD40L is primarily derived from T cells and activated platelets (74). Soluble CD40L within platelets mediates thrombogenesis and inflammatory responses, and its levels are closely associated with an increased risk of inflammation and cardiovascular diseases related to viral infections (29). Platelet-derived sCD40L promotes CD40-positive cell activation and thrombogenesis by stabilizing integrin αIIbβ3 in COVID-19. Compared to healthy controls, COVID-19 patients show elevated sCD40L levels, which decline over time as a potential biomarker for inflammatory monitoring (75, 76). CD40 also plays a pivotal role within the central nervous system, where it is expressed in astrocytes, microglia, and vascular endothelial cells. CD40 on microglia can interact with CD40L on infiltrating T lymphocytes or other cells within the CNS, triggering intracellular signaling events and culminating in the production of a plethora of cytokines and neurotoxins (77). The level of circulating sCD40L reflects the activation status of the CD40-CD40L complex. Platelet-expressed CD40L serves as a pivotal inflammatory mediator, triggering the activation of astrocytes and microglia, a process that is intricately linked to the activation of mitogen-activated protein kinases (MAPKs), degradation of IκB-α, and the NFκB-mediated inflammatory signaling cascade. The resultant indices of glial activation may ultimately precipitate platelet aggregation, neuroinflammatory responses, and neuronal damage (78). Elevated levels of soluble CD40L are observed in the plasma of patients with inflammatory demyelinating diseases, and sCD40L has also been implicated in the disruption of the blood-brain barrier (79–81). Moreover, existing evidence suggests that the concentration of sCD40L is independently associated with an increased risk of radiological progression in cerebral small vessel disease (82). The aforementioned mechanisms of abnormal platelet deposition, neuroinflammation, and disruption of the blood-brain barrier may be one of the reasons by which SARS-CoV-2 affects CSVD through the CD40 and CD40L.

sTREM-2, a cell surface receptor and a member of the Triggering Receptors Expressed on Myeloid cells (TREM) family, is associated with the induction of type 2 immune responses following viral infections. There is evidence to suggest that the plasma concentration of sTREM-2 may be a potential independent predictor of severe disease in COVID-19 patients, which could play a crucial role in distinguishing the severity of the disease (83). TREM-2, a receptor with the capacity to discern pathogen-associated molecular patterns, has been found to engage with the M protein of SARS-CoV-2 via its extracellular immunoglobulin (Ig) domain. This interaction triggers a signaling cascade, culminating in the activation of T cells and the subsequent augmentation of pro-inflammatory cytokine production by T helper 1 (TH1) cells, including interferon-β and tumor necrosis factor. The resultant cytokine release intensifies the inflammatory response and contributes to tissue injury (30). Within the brain, the immune receptor TREM2 can be expressed in microglia. TREM2 regulates microglial proliferation and survival by activating the Wnt/β-catenin signaling pathway. Additionally, TREM2 can also activate the Akt/β-catenin pathway to promote microglial proliferation and survival following injury (84). The levels of sTREM2 in peripheral blood and cerebrospinal fluid (CSF) are closely correlated (85), an elevation in the levels of soluble TREM2 within the cerebrospinal fluid has been observed to positively correlate with the progression of CSVD, particularly in conjunction with the imaging biomarker of cerebral microbleeds. Plasma levels of soluble TREM2 have been implicated as a predictive biomarker for white matter injury associated with small vessel pathologies. The therapeutic targeting of sTREM2, particularly in the CSF, may hold significant clinical implications for the management of CSVD (86). It is, however, imperative that the relationship between cerebral small vessel disease and the aforementioned biomarkers be substantiated through more comprehensive research, especially in the context of prolonged COVID-19 infection, the concurrent validation of the effectiveness of intervention strategies and the determination of the optimal timing for such interventions are also critical issues that require resolution.

### 2.3 The oxidative stress elicited by COVID-19 infection may serve as a contributing factor to the development or progression of CSVD

In patients with COVID-19, in addition to the observed impacts of the aforementioned inflammatory responses, there is also an enhancement of oxidative stress. Long COVID patients exhibit elevated oxidative damage driven by reactive oxygen species (ROS), while oxidative stress pathways drive endothelial dysfunction and amplify inflammatory responses. (31, 87). NADPH oxidase enzymes, denoted as NOX, are the producers of reactive oxygen species, it has been documented that knocking down Nox1, a member of the NADPH oxidase family known for generating reactive oxygen species, in rats can reduce ROS production and subsequently improve cognitive impairment. Moreover, Nox2, another member of this family, has been implicated in the induction of blood-brain barrier disruption and vasomotor dysfunction under ischemic conditions (88–90). In the context of focal cerebral ischemia, mice with a deficiency in Nox4, yet another isoform of the NADPH oxidase family, exhibit reduced brain injury, underscoring the pivotal role of NOX4-derived oxidative stress in the pathophysiology of acute ischemic stroke (91). Oxidative stress has been implicated in the etiology of cerebral small vessel disease, encompassing both non-amyloidogenic and amyloidogenic subtypes, and is posited to contribute to vascular damage and cognitive dysfunction (92). Consequently, oxidative stress may also be considered a contributing factor to COVID-19-induced CSVD.

### 2.4 SARS-CoV-2 may exert its impact on CSVD by cleaving the nuclear factor (NF)-κB essential modulator (NEMO) through the action of its 3C-like protease

The NF-κB essential modulator is a human immunological signaling protein that, under physiological conditions, activates NF-κB within the canonical NF-κB response signaling pathway, which is a pivotal immune response against viral infections. NEMO can be cleaved by the non-structural protein 3C-like protease encoded by SARS-CoV-2, leading to the inhibition of host immune responses and contributing to the severe consequences of COVID-19 infection. The cleavage of NEMO by 3CLpro results in the disruption of the NF-κB pathway, a hallmark of chronic inflammatory diseases, suggesting that COVID-19 infection may have long-lasting effects on the human body (32, 93). Jan Wenzel and colleagues, through a series of rigorous experiments, ultimately found that the toxic effects of 3CLpro are mediated by its protease activity and the cleavage of NEMO, with capillaries being particularly vulnerable to the impact of NEMO deficiency. Furthermore, the authors observed that the absence of RIPK3 or the inhibition of RIPK1 can prevent NEMO ablation, thereby contributing to the amelioration of microvascular pathology (33). A study focusing on the genetic disorder incontinentia pigmenti (IP), which shares features with CSVD, similarly implicates NEMO as a critical component of the NF-κB signaling pathway. When NEMO is mutated and rendered inactive, IP can be induced. NEMO is indispensable for angiogenesis; mice subjected to continuous NEMO ablation to disrupt angiogenesis exhibit severe functional deficits. Compared to endothelial cells with NEMO deficiency, those with normal NEMO function exhibit higher proliferation rates. In this CSVD model, NEMO-associated angiogenesis can counteract functional deficits and improve the condition (94). The mechanisms by which COVID-19 infection leads to cerebral microvascular pathology may be related to CSVD, and therefore, reducing NEMO inactivation may confer benefits to COVID-19-infected patients by mitigating neurological complications.

### 2.5 SARS-CoV-2 exerts its influence on CSVD by compromising neurovascular coupling

The intricate developmental, structural, and functional interplay between brain cells and the microvascular system, as well as their coordinated response to injury, is realized through the neurovascular unit (NVU) (95). The induction and maintenance of the blood-brain barrier are also closely intertwined with the NVU, with astrocytes and pericytes being key constituents that significantly impact the BBB’s normal function (96). Among the various aspects of the NVU, neurovascular coupling has been a subject of extensive research. NVC refers to the coupling between neuronal activity and the vascular system, which is orchestrated by a series of highly coordinated multicellular interactions. NVC plays a crucial role in regulating cerebral blood flow (CBF) and neuronal activity, and it also influences cognitive dysfunction (97). There is a highly consistent association between CSVD and impaired NVC, particularly in relation to white matter hyperintensities, where endothelial dysfunction plays a central role in CSVD. NVC holds promise as a clinical method for assessing disease progression and treatment response in CSVD (98). Existing evidence suggests that SARS-CoV-2 infection can induce NVC damage, leading to persistent cognitive impairment in patients. The post-infection increase in reactive oxygen species generation mediated by AT1R and AngII-induced ROS production in brain microvascular endothelium activates transcription factors (e.g., nuclear factor κB), thereby increasing the synthesis of pro-inflammatory mediators and adhesion molecules. This interplay between oxidative stress and inflammation disrupts NVC and leads to endothelial dysfunction (34). SARS-CoV-2 may potentially cause endothelial dysfunction and long-term cognitive dysfunction by disrupting NVC. Given that cognitive impairment is a prominent symptom in CSVD, it is plausible that COVID-19 may contribute to the development and progression of cerebral small vessel disease by damaging NVC.

### 2.6 SARS-CoV-2 impacts CSVD by means of circulating microparticles

Microparticles, cell-derived vesicles measuring between 0.1 and 1 μm in diameter, have garnered increasing recognition in the scientific community. These MPs originate from the plasma membranes of endothelial cells, platelets, leukocytes, and erythrocytes, with their formation being modulated by intracellular calcium signaling pathways, αIIβ3 integrin, and the turnover of the cytoskeleton (99). MPs are facilely transported within the vascular system, capable of conveying pro-inflammatory signals to adjacent or target cells, thereby functioning as robust carriers of biomolecular information and as pivotal mediators of intercellular communication (100, 101). Extensive research has demonstrated that elevated levels of MPs can be observed in patients suffering from acute coronary syndrome, stroke, diabetes, pulmonary and systemic hypertension, as well as hypertriglyceridemia (102), with diabetes and hyperlipidemia being potential risk factors for cerebral small vessel disease. In patients with CSVD, it has been noted that individuals with white matter hyperintensities (WMH) exhibit a marked increase in platelet-derived MPs (PDMPs), leukocyte-derived MPs (LMPs), and total MP count. Elevated MP levels in symptomatic patients may signify underlying microvascular occlusion, suggesting that circulating MPs could serve as a novel surrogate marker for white matter integrity in CVSD (103). COVID-19 infection promotes microvesicle (MP) formation, which enhances coagulation, thrombosis, and inflammation through multiple mechanisms. These MPs may drive CSVD progression with prolonged effects persisting post-pandemic, leading to occult CSVD. During the cytokine release syndrome (CRS) phase of SARS-CoV-2 infection, membrane remodeling exposes procoagulant phosphatidylserine (PS), while TNF-α may also induce ACE2-bearing microvesicle release from microvascular endothelial cells. MPs shed through these pathways may cause capillary endothelial dysfunction and microcirculatory disruption, with ACE2-carrying MPs potentially forming lung-to-brain emboli that deposit in cerebral tissues. Furthermore, MPs elevate pro-inflammatory cytokine levels (IL-1, IL-6, IL-8, and TNF-α). (Che Mohd 103–105). A potential vicious cycle between these processes provides a plausible hypothesis for SARS-CoV-2-induced CSVD.

## 3 The Human Immunodeficiency Virus

Human Immunodeficiency Virus type 1 (HIV-1) is an enveloped retrovirus characterized by a conical core encapsulating the viral genome, which acquires a lipid membrane through its aggregation at the plasma membrane of the infected cell (106). The structural polyprotein Gag, primarily responsible for viral assembly, is composed of four structural domains (matrix (MA), capsid (CA), nucleocapsid (NC), and p6), as well as two short spacer peptides, SP1 and SP2 (107). The replication process of HIV-1 is highly complex, encompassing early stages such as viral binding to cell surface receptors, entry into the cell, reverse transcription of viral RNA to DNA, nuclear import, and integration of viral DNA. The late phase of replication involves the translation of viral RNA to produce Gag polyprotein precursors, GagPol polyprotein precursors, envelope glycoproteins (Env glycoproteins), and regulatory and accessory viral proteins, culminating in the complete process from gene expression to the release and maturation of new viral particles (108). HIV targets cells expressing the CD4 receptor and the chemokine receptors CCR5 and CXCR4, thereby leading to systemic T cell destruction and immunodeficiency through the aforementioned cellular invasion process. Additionally, HIV infection via monocytes can inflict damage on the gut, lungs, and brain, contributing to chronic cardiovascular, hepatic, pulmonary, and central nervous system diseases through its effects on immunity and the endothelium (109). The HIV pandemic persists as a predominant global public health issue, distinguished by its genetic diversity. The distribution of HIV-1 subtypes and recombinants is dynamically evolving across various countries and regions (110). Despite the widespread adoption of antiretroviral therapy, which has partially reduced the risk of mortality and new infections, ongoing vigilance is required to address the adverse impacts of HIV and to refine its prevention and control strategies.

### 3.1 HIV-associated neurocognitive disorders and CSVD

HIV-associated neurocognitive disorders are particularly pronounced among HIV-related comorbidities, adversely affecting patients’ quality of life. Cerebral small vessel disease is a significant cause of cognitive impairment, leading us to speculate that CSVD may be an important factor contributing to HAND. HIV infection is associated with increased white matter hyperintensity burden, as HIV-positive individuals exhibit higher WMH levels than controls (111). Among HIV-positive patients with microalbuminuria, those who received combined antiretroviral therapy (cART) demonstrate impaired information processing speed, which may correlate with cerebrovascular small vessel disease. Microalbuminuria, as a rapid and inexpensive screening method, may become a model for assessing cognitive function in resource-limited countries (112). The emergence of these CSVD-related clinical symptoms and imaging changes in HIV patients highlights the importance of exploring the mechanisms by which HIV infection causes CSVD.

### 3.2 The potential mechanisms of combined antiretroviral therapy leading to CSVD

Despite the continuous advancement of combined antiretroviral therapy, a significant proportion of chronically HIV-infected individuals—ranging from 18% to 50%—still develop HIV-associated neurocognitive disorders (113). Among middle-aged individuals, even with sustained immunovirological control, the prevalence of asymptomatic cerebral small vessel disease in infected individuals is found to be twice that of their uninfected counterparts (114). Certain components of highly active antiretroviral therapy (HAART) based on protease inhibitors (PIs) may exert toxic effects on the cerebral microvascular endothelial and smooth muscle cells, leading to vascular wall degeneration. Additionally, these medications may indirectly increase the risk of CSVD and exacerbate cognitive impairment by inducing metabolic abnormalities such as dyslipidemia and insulin resistance (36). However, it remains unclear whether HIV and its associated treatments invariably induce neurocognitive dysfunction. Some studies have found no adverse effects of antiretroviral therapy class exposure on CSVD in treated middle-aged HIV-infected individuals, suggesting that HIV infection and CSVD may be independent processes that cumulatively contribute to cognitive impairment (115, 116).

### 3.3 HIV is likely to affect CSVD through the mechanism involving neopterin

Neopterin, a pyrazine-pyrimidine compound produced by cells of the monocyte-macrophage lineage and astrocytes, functions in response to interferon-γ stimulation and may serve as a biomarker for HIV-associated central nervous system damage (37, 117). Neopterin is closely associated with monocytes, the activation of which has been linked to cognitive decline in HIV+ individuals (118), and it is plausible that these monocytes may migrate into the brain and exert influence. Furthermore, neopterin can activate nuclear factor-κB, enhancing the expression of adhesion molecules (119). Endothelial activation drives blood-brain barrier (BBB) disruption and neuroinflammation, contributing to small vessel damage in CSVD. Neopterin levels are elevated in CSVD patients compared to non-CSVD individuals (120), suggesting its potential as a biomarker for HIV-associated CSVD.

### 3.4 CD163 may serve as an explanatory factor for the impact of HIV on CSVD

The scavenger receptor CD163, expressed by monocytes and macrophages, upon shedding, transforms into its soluble form, sCD163 (121). sCD163 serves as a biomarker of HIV activity, linking viral replication to monocyte and macrophage activation. (38). CD163 may also play a role in the pathomechanisms of cerebral small vessel disease. Cerebral autosomal dominant arteriopathy with subcortical infarcts and leukoencephalopathy (CADASIL), a subtype of CSVD, has been investigated through comparative analysis of the inflammatory and immune responses in CADASIL patients versus controls, revealing a pronounced accumulation of microglia/macrophages around microvessels.CD163-positive cells were associated with a specific perivascular inflammatory cell response (122). In early hypertension, detailed analysis of cellular subpopulations identified a subpopulation of microglia expressing CD163, with altered microglial function implicated in blood-brain barrier leakage and responsiveness to vascular dysfunction, factors that may collectively contribute to the progression of CSVD (123).

### 3.5 HIV might influence CSVD through the mediation of high-mobility group box 1

High-mobility group box-1 protein, a nuclear factor and secreted protein, is implicated in the maintenance of the nucleosome structure as well as in DNA replication, transcription, and recombination (124, 125). HMGB1 can be actively secreted during stress and passively released by damaged or necrotic cells, engaging in the production of pro-inflammatory cytokines (126). High HMGB1 levels in plasma correlate with viral loads in HIV-1 infection, suggesting that HMGB1-containing immune complexes may participate in the pathogenesis of HIV-1 (39). Concurrently, HMGB1 emerges as a risk factor for cognitive impairment in patients with CSVD, as increased levels of HMGB1 promote the activation of microglia, leading to a sustained inflammatory response that disrupts the neurovascular unit and the blood-brain barrier, resulting in neurodegenerative necrosis and ultimately cognitive impairment and cerebral microbleeds (127). This may account for the cognitive decline observed in HIV patients and those afflicted with CSVD.

## 4 Hepatitis C virus

The Hepatitis C virus, a member of the Hepacivirus genus within the Flaviviridae family, is characterized as a small, enveloped, single-stranded RNA virus (128). The HCV genome comprises a positive-sense single-stranded RNA of approximately 9.6 kilobases, housing an open reading frame that encodes a polyprotein precursor consisting of about 3000 amino acid residues, flanked by 5’ and 3’ non-translated regions (NTRs) (129). The polyprotein encoded by the viral genome is proteolytically cleaved by both cellular and virus-encoded proteases to generate at least ten distinct mature viral proteins, including structural and non-structural proteins that are critical for the normal functioning of HCV (128). The HCV virion is encapsulated within an icosahedral capsid, and the virus lifecycle is initiated subsequent to attachment to specific receptors (130). The viral RNA, upon its entry into the cytoplasm, undergoes translation through an internal ribosome entry site (IRES) located within the untranslated regions. This process of RNA translation occurs on the rough endoplasmic reticulum (ER), subsequent to which the nascent viral particles undergo processing and are then expelled into the extracellular milieu via exocytosis (131), thereby initiating the adverse effects on the human body. HCV is primarily transmitted through parenteral routes, with the illicit use of injectable drugs and high-risk sexual practices significantly facilitating viral dissemination. HCV exhibits a prolonged incubation period, with acute infection typically being asymptomatic; chronic HCV infection can progress from chronic hepatitis to more severe conditions such as liver cirrhosis and hepatocellular carcinoma (132).

### 4.1 HCV and cerebral small vessel disease

Hepatitis C virus infection can manifest a variety of extrahepatic manifestations, which diminish the quality of life of patients and augment their economic and health burdens (133–135). HCV infection may precipitate acute and subacute involvement of white matter, elicit inflammatory diseases of the central nervous system, and present with cognitive impairment, alterations in consciousness, as well as sensory and motor dysfunction (136). By virtue of viral localization within the lesions of HCV seropositive patients’ plaques, it has been observed that HCV infection factors might penetrate target cells through LDL receptors or scavenger receptor B1, thereby enhancing lipoprotein oxidation. The local effects of this process are believed to play a role in the pathogenesis of carotid atherosclerosis (137), potentially influencing cerebrovascular function as a consequence. Chronic hepatitis C virus infection correlates with cerebrovascular lesion development, supported by evidence that elevated serum HCV RNA levels associate with increased cerebrovascular mortality risk (138). However, the literature on the relationship between HCV and cerebral small vessel disease is limited, prompting us to investigate this association. Both HCV and HIV infections can present with mild neurocognitive impairment, exhibiting similar patterns of injury. While these diseases may share a common pathogenic mechanism, co-infected HIV and HCV patients exhibit poorer cognitive function than those with HIV alone. (139). As a common comorbidity of HIV, HCV infection not only results in more severe cognitive impairment but also manifests with higher HIV RNA levels and increased levels of MCP-1, an inflammatory chemokine, in cerebrospinal fluid (140). In light of the presence of cognitive impairment and inflammation, we postulate that HCV may induce the occurrence and progression of CSVD through a unique mechanism.

### 4.2 Possible mechanisms by which HCV affects CSVD

Cultured HCV infects brain microvascular endothelial cells (BMECs), which express HCV entry-required factors and mediate viral entry and replication. This infection increases endothelial permeability, induces apoptosis in BMECs, and damages the blood-brain barrier. (40, 41). These phenomena are linked to cerebral small vessel disease. A tissue-based study of brain small artery disease identifies HCV as an independent CSVD risk factor. HCV-induced small artery injury may arise from lipid/glucose metabolic disorders, elevated inflammatory burden, and endothelial dysfunction. (141). Moreover, HCV may increase the levels of CXCL10 released by HBMECs through the phosphorylation of NF-κB. The receptor for CXCL10 is CXCR3, which ultimately leads to the recruitment of CXCR3-positive leukocytes to the damaged central nervous system, thereby affecting it. Consequently, inhibiting lymphocyte migration might mitigate the harm caused by neuroinflammatory diseases, potentially intervening in the development of CSVD (42).

## 5 Zika virus

Zika virus is a single-stranded positive-sense RNA virus that belongs to the genus Flavivirus within the family Flaviviridae, characterized by enveloped icosahedral virions measuring 40 to 50 nanometers in diameter (142). The viral genome is flanked by non-coding regions, with an open reading frame (ORF) that encodes three structural proteins essential for the assembly of viral particles, as well as seven non-structural proteins that facilitate genome replication and packaging (143). Surface receptors on host cells, such as the AXL family of receptor tyrosine kinases and C-type lectins, are likely involved in the interaction with viral surface glycoproteins, initiating viral RNA replication and translation, and completing the assembly of viral particles in the endoplasmic reticulum (ER), ultimately leading to the release of virions from the host cell (144). Zika virus is primarily transmitted by mosquitoes or ticks, and can also be spread through urine, blood transfusions, mother-to-child transmission, as well as sexually. The infection caused by ZIKV has spread globally, hence it is imperative to recognize the potential threat posed by ZIKV to global public health (145). Upon the onset of the febrile phase in the course of ZIKV infection, viral RNA is detectable in serum specimens through reverse transcription polymerase chain reaction (RT-PCR) within the early post-infection period. ELISA tests for ZIKV-specific IgM and IgG have also contributed to the diagnostic process of the disease (146). Infected individuals may present with symptoms such as fever, rash, arthralgia and myalgia, conjunctivitis, and headache. Zika virus infection has also been associated with severe illnesses, including meningitis, encephalitis, and thrombocytopenia (147). Notably, there has been an increased incidence of Guillain-Barré syndrome (GBS) in adults associated with ZIKV infection, indicating a close correlation between ZIKV and central nervous system diseases (148, 149).

### 5.1 Zika virus and CSVD

Following the acute phase of ZIKV infection, the virus can persist, indicating that it may have established an equilibrium for cell survival and viral replication within cellular reservoirs, while also exhibiting the capacity to evade both innate and adaptive immune responses. Human brain microvascular endothelial cells (hBMECs) act as reservoirs for ZIKV, enabling basolateral release into neurons and causing chronic neurological damage. Prolonged persistence in hBMECs promotes cerebrovascular small vessel disease (150).

### 5.2 Possible mechanisms of CSVD induced by Zika virus

ZIKV infection of endothelial cells, pericytes, and astrocytes in the blood-brain barrier may contribute to CSVD pathogenesis by disrupting BBB integrity. Additionally, ZIKV is capable of upregulating the levels of inflammatory cytokines (IL-6 and IL-8), chemokines (CCL5 and CXCL10), and cell adhesion molecules (CAMs), ultimately resulting in immune cell infiltration and neuroinflammation within the central nervous system (151). ZIKV can also infect monocytes in the circulation, enhancing their adhesive and migratory capabilities, and thereby facilitating their recruitment to the CNS (43). Furthermore, the non-structural protein NS1 of ZIKV influences the adherens junction proteins crucial to the endothelial barrier of human brain microvascular endothelial cells through the hsa-miR-29b-3p/DNMT3b/MMP-9 pathway, thereby compromising the barrier function of human cerebral vascular endothelial cells (44). These alterations may also be manifest in the pathological changes of CSVD, including the disruption of the BBB, the upregulation of inflammatory mediators, and the infiltration of inflammatory cells.

### 5.3 The Zika virus could potentially influence CSVD via sodium-dependent lysophosphatidylcholine symporter 1

The sodium-dependent lysophosphatidylcholine symporter, known as Mfsd2a, serves as a membrane transport protein and is primarily expressed in the endothelial cells of the blood-brain barrier (152). In experimental research, Jia Zhou and colleagues demonstrated that ZIKV glycoprotein E interacts with Mfsd2a to promote its ubiquitination, causing BBB disruption and establishing a mechanistic link between Mfsd2a and ZIKV-induced neurovascular abnormalities (45). Current reports suggest a decline in cognitive function during the acute phase of ZIKV infection (153, 154). Despite the lack of long-term, large-scale follow-up studies on cognitive outcomes post-infection and direct evidence linking ZIKV to cerebrovascular small vessel disease, the prolonged replication of ZIKV could potentially impair BBB integrity through the ubiquitination of Mfsd2a, resulting in cognitive decline and the induction of CSVD.

## 6 Syphilis

Syphilis is an infection caused by Treponema pallidum subspecies pallidum, which can be transmitted through sexual contact or during pregnancy to the fetus, leading to congenital syphilis (155). The complete genome sequence of Treponema pallidum (TPA) reveals a circular chromosome comprising approximately 1,138,006 base pairs. The low protein content in the outer membrane of TPA may be a key factor in its immune evasion strategy (156). TPA is an obligate human pathogen. The clinical course of human infection with TPA is divided into early syphilis, which includes primary, secondary, and early latent syphilis, and late syphilis, encompassing late latent syphilis and tertiary syphilis (157). Primary syphilis is characterized by the appearance of chancres at the site of infection, secondary syphilis may present with skin and mucosal lesions, rashes, and lymphadenopathy; latent syphilis is not transmissible sexually, and tertiary syphilis includes gummatous syphilis, late cardiovascular syphilis, and late neurosyphilis (158).

### 6.1 Syphilis and CSVD

Patients with early neurosyphilis may exhibit symptoms such as altered mental status, cranial nerve involvement, motor and sensory deficits, meningitis, or stroke. The clinical manifestations of late neurosyphilis may include progressive cognitive dysfunction, sensory deficits, gait abnormalities, and severe radicular pain (159). In addition to these symptoms, syphilis infection may also increase the risk of ischemic stroke in patients, although the exact mechanism remains elusive. Vascular inflammation leading to stenosis or occlusion of blood vessels is a convincing explanation; a retrospective study revealed that among patients with ischemic stroke, those with positive syphilis serology frequently had intracranial arterial stenosis. Poorly controlled syphilis infection may be closely associated with intracranial arterial stenosis (160), suggesting that syphilis infection may have potential mechanisms that affect the intracranial vasculature. A study conducted a follow-up on neurosyphilis patients diagnosed with acute ischemic stroke and ultimately found that, compared to patients without syphilis infection, those with neurosyphilis exhibited a closer relationship with the imaging manifestations of cerebral small vessel disease and lower cognitive function scores (161). Therefore, syphilis infection may be associated with CSVD through a variety of mechanisms.

### 6.2 The possible mechanisms through which syphilis induces CSVD

Firstly, cerebrospinal fluid analysis in syphilis patients may reveal lymphocytosis or elevated protein concentrations, suggesting the presence of neurological infiltration (162). Secondly, lipoprotein Tp0751, as one of the complex pathogenic proteins of Treponema pallidum, may affect cerebral vascular endothelial cells through the MAPK pathway and the NF-κB pathway, and Tp0751 can stimulate the production of IL-6 in cerebral vascular endothelial cells, thereby disrupting the tight junction proteins in the blood-brain barrier (46). The aforementioned disruption of the blood-brain barrier, neurological infiltration, and upregulation of inflammatory factors may also link syphilis to CSVD. Neurofilament light subunit (NF-L) and phosphorylated neurofilament heavy subunit (pNF-H) serve as biomarkers for assessing the extent of neuronal damage in neurodegenerative diseases. In patients with symptomatic neurosyphilis, elevated concentrations of NF-L and pNF-H in cerebrospinal fluid have been observed, and their levels significantly decreased following treatment (47). Elevated blood NfL levels correlate with CSVD severity and serve as a marker for disease burden. (163). Moreover, axonal pNfH is typically concentrated around small penetrating arteries, which corresponds to the location of CSVD arterial lesions, potentially representing the result of subcortical white matter axonal damage due to CSVD arterial pathology (164). Consequently, NF-L and pNF-H hold promise as biomarkers for assessing the burden of CSVD in patients with syphilis.

## 7 Gut microbiota

The gut, as the largest digestive organ in the human body, possesses a complex and diverse microbial community due to its unique structure that interfaces with the external environment (165). This community comprises bacteria, fungi, protozoa, archaea, and viruses, with an estimated 100 trillion bacteria populating the intestinal tract, including over 1500 species across approximately 50 genera. Bacteria dominate the gut microbiota, with more than 90% belonging to the phyla Bacteroidetes and Firmicutes (166). The gut microbiota exhibits individual variations influenced by geography, environment, diet, age, genetics, disease, and lifestyle (167). The intestinal microbiome confers a range of beneficial functions to the human body, playing a crucial role in shaping the gut immune system, enhancing the metabolic capabilities of the intestine, providing essential nutrients to the host, and inhibiting the colonization of exogenous pathogens (168). An imbalance in the gut microbiota composition can trigger modifications in physiological processes through multiple pathways. It may contribute to the onset of various diseases, with the gut microbiome playing a pivotal role in the progression of these illnesses (169). Beyond the digestive system, such dysbiosis may also impact the central nervous system, leading researchers to conceptualize the microbiota-gut-brain axis—a communicative bridge comprising the central nervous system, enteric nervous system (ENS), hypothalamic-pituitary-adrenal axis, gut, and microbiota (170). The interaction between the gut microbiota and the central nervous system occurs via several pathways, including the gut microbiota and their metabolites, the intestinal immune system, the ENS, and the neuroendocrine system (171). For instance, the gut microbiota can communicate directly with the central nervous system via the vagus nerve, regulate the expression of tight junction proteins to indirectly modulate the transport of certain molecules across the blood-brain barrier, and compounds such as gallic acid, trimethylamine-N-oxide (TMAO), and other microbial metabolites have been demonstrated to traverse the BBB, substantiating the intimate connection between the gut microbiota and the central nervous system (172).

### 7.1 Gut microbiota and CSVD

It is currently established that dysbiosis of the gut microbiota can elevate the risk of cerebrovascular diseases through diverse mechanisms (173). These include immune activation, lipid dysregulation, and platelet hyperactivity (mediated by TMAO, PAGln, and PAGly), which not only trigger atherosclerosis but also exacerbate cerebrovascular disease. (174). Furthermore, there is a correlation between the intricate composition of the gut microbiota and the observed decline in cognitive abilities among middle-aged and elderly individuals. Consequently, investigating the interplay between the gut microbiota and cerebral small vessel disease assumes significant clinical relevance.

### 7.2 Potential mechanisms by which the gut microbiota induces CSVD

In an animal study, it was observed that the gut microbiota might contribute to the transformation of inflammation from a localized intestinal condition to a systemic inflammatory response through the actions of bacterial toxins and the translocation of bacteria themselves, leading to invasion of the brain and disruption of the blood-brain barrier. The loss of BBB integrity is frequently detected in the early stages of cerebral small vessel disease (175). A study linking gut microbiota to CSVD patients reveals a significant correlation between *Barnesiella intestinihominis* and CSVD biomarkers. Differences in bacterial gene abundance associate with molecules including AMP/GMP-activated kinases, xanthine, tyrosine, phenylpyruvate, and dicarboxylic acid-hydroxybutyrates—compounds implicated in neurodegenerative diseases. Notably, AMP-activated kinases and hydroxybutyrates demonstrate neuroinflammatory suppressive effects, suggesting that gut microbiota-CSVD associations may be regulated via neuroinflammatory pathways (48). Parasutterella, a pivotal genus within the human gut microbiota, has been demonstrated to exhibit a significant correlation with cognitive assessments such as the Mini-Mental State Examination (MMSE) and the Montreal Cognitive Assessment (MoCA) in patients with cerebral small vessel disease. Additionally, the relative abundance of Parasutterella is associated with the amplitude of low-frequency fluctuation (ALFF) within the bilateral middle frontal gyrus, these findings suggest a potential contribution of Parasutterella to the etiology of cognitive dysfunction in CSVD. Moreover, the relative abundance of Parasutterella is positively associated with plasma levels of S100β, a peripheral marker of blood-brain barrier functionality, suggesting that Parasutterella may contribute to the degradation of BBB integrity in the context of CSVD. The study also reports a positive correlation between the levels of the gut bacterium Collinsella in CSVD patients and white matter hyperintensity scores, with an increase in Collinsella being linked to inflammatory activity and tumor necrosis factor-alpha (TNF-α) levels, which may participate in neuroinflammatory responses and reflect the severity of CSVD (49).

#### 7.2.1 Gut microbiota and arteriosclerotic cerebral small vessel disease

Disruption of the gut microbiota is now widely acknowledged to be significantly associated with neuroinflammation and cognitive dysfunction (176). A study on arteriosclerotic cerebral small vessel disease (aCSVD) revealed that alterations in the gut microbiota can influence inflammatory responses. The aCSVD-associated gut microbiota, through RORγt signaling, activates neutrophils leading to upregulated IL-17A expression. During gut microbiota dysbiosis, the pro-inflammatory characteristics of neutrophils are enhanced, with increased expression of IL-17A, compromising the blood-brain barrier and facilitating further neutrophil infiltration into the brain. The pathological mechanism involves the gut microbiota-immune system-brain axis, which may play a crucial role in the progression of aCSVD (50).

#### 7.2.2 Gut microbiota and cerebral autosomal dominant arteriopathy with subcortical infarcts and leukoencephalopathy

Cerebral autosomal dominant arteriopathy with subcortical infarcts and leukoencephalopathy (CADASIL), a form of cerebral small vessel disease, although the presence of a genetic mutation is a prerequisite, the impact of environmental factors on its pathogenesis cannot be overlooked. CADASIL patients exhibit reduced levels of probiotics Eubacterium eligens and Roseburia faecis, which modulate local/systemic inflammation to influence disease progression. Gamma-aminobutyric acid (GABA), a critical inhibitory neurotransmitter essential for neural function maintenance and neuronal excitability suppression, is accompanied by elevated abundance of GABA-consuming bacteria (Megasphaera elsdenii, Eubacterium siraeum), which disrupt GABA-mediated inhibition and drive neurotransmitter imbalance. Moreover, the gut microbiota’s Candida albicans can activate the non-canonical caspase-8-dependent inflammasome in macrophages, thereby upregulating IL-1βand promoting neuroinflammation. These mechanisms may all be involved in the onset and development of CADASIL (51).

## 8 Oral microbiota

In the distinctive oral environment of the human body, over 700 species of microorganisms, including a wide array of bacteria, viruses, archaea, fungi, and protozoans, have established colonization. The oral microbiota acts as a mirror, reflecting the physiological status of the human body, and possesses unique value in predicting disease risk and therapeutic efficacy, particularly in instances of oral microbiota dysbiosis (177, 178). Various oral microorganisms are potential triggers of dental caries and periodontitis, and their prevalence is substantially regulated by host factors such as diet and inflammation (179). Oral bacteria can translocate to distal sites and may also induce bacteremia and systemic dissemination of oral bacteria through the disruption of the periodontal epithelial barrier, thereby facilitating the pathogenesis of diseases (180, 181). Although this type of bacteremia is typically transient, the pro-inflammatory and immunomodulatory effects exerted by the disseminated oral bacteria upon reaching distal organs, such as the brain, bone marrow, cardiovascular tissues, and liver, can be significant (182). Current research indicates a close association between certain oral microorganisms and the pathogenesis of multiple sclerosis, Alzheimer’s disease, and Parkinson’s disease (PD) (183–185). Investigations into Parkinson’s disease have revealed that the Kgp II genotype of Porphyromonas gingivalis is correlated with the cognitive impairment observed in affected patients (186). Periodontal bacteria-induced pro-inflammatory cytokines may enter brain tissue via systemic circulation or peripheral nerves (e.g., trigeminal/glossopharyngeal), thus triggering CNS disorders (178).

### 8.1 Oral bacteria and CSVD

There is a close association between oral bacteria and periodontal disease, with current knowledge suggesting that periodontal disease may induce cerebral small vessel disease, and lacunar infarction may be related to periodontal disease. Moreover, the progression of periodontal disease often portends an increase in the number of lacunar infarctions (187). Despite adjusting for risk factors of lacunar stroke, chronic periodontitis remains independently associated with the presence of lacunar infarcts (188). Chronic periodontal disease is considered to potentially induce a chronic systemic inflammatory response and endothelial dysfunction (189). Chronic periodontitis leads to the production of a significant amount of circulating pro-inflammatory mediators (IL-1β, IL-6, TNFα), C-reactive protein (CRP), etc. (190–192). It is known that inflammatory factors such as IL-6, TNFα, and CRP are associated with an increased risk of cognitive decline in patients with cerebral small vessel disease (193). The amassing of a substantial quantity of inflammatory mediators could potentially offer a mechanistic nexus between periodontitis and cerebral small vessel disease, this prompted us to explore additional clues.

### 8.2 Possible mechanisms of Porphyromonas gingivalis inducing CSVD

Porphyromonas gingivalis, a member of the oral microbiota, directly infects human endothelial cells, activates p38 MAPK phosphorylation and NF-κB nuclear translocation, and enhances endothelial adhesion molecule expression, thereby inducing endothelial damage and inflammatory responses. (52). Furthermore, the bacterial-induced autoimmune response may also result in endothelial injury, as the interaction between oral bacterial antigens and endogenous molecular structures may elicit an autoimmune response; the immunological cross-reaction of antibodies and T cells between endogenous heat shock proteins and the molecular chaperone factor GroEL of Porphyromonas gingivalis may lead to endothelial dysfunction, ultimately triggering cerebral small vessel disease (53). In addition to endothelial damage, the abnormal aggregation of platelets is also of note, as the cysteine protease produced by Porphyromonas gingivalis can activate protease-activated receptors (PAR)-1 and -4 expressed on the platelet surface, ultimately causing abnormal platelet aggregation (54), which may affect the small vessels of the brain. Moreover, Porphyromonas gingivalis is present in atherosclerotic plaques, and systemic exposure to this bacterium may increase the risk of ischemic stroke (194). Intracranial atherosclerosis is also crucial for patients with CSVD (195).

### 8.3 The potential mechanism by which CNM-positive *Streptococcus mutans* induces CSVD

CNM is a collagen-binding protein located on the cell surface of *Streptococcus mutans*. The presence of CNM-positive *S. mutans* in the oral cavity is strongly linked to severe dental caries. Infection of carious teeth with *S. mutans* raises the risk of bacteremia and cerebral dissemination. Additionally, *S. mutans* adheres to tooth surfaces, and even routine activities like brushing or flossing may trigger bacteremia. Once in the bloodstream, CNM-positive *S. mutans* induces blood-brain barrier inflammation by binding to cerebral vascular basal membranes (BM), causing cerebrovascular damage (55). A population-based study revealed that individuals infected with collagen-binding protein-positive Streptococcus mutans exhibited a higher incidence of cerebral microbleeds, suggesting that CNM-positive Streptococcus mutans may elevate the risk of cerebral microbleeds and, consequently, demonstrate an association with cerebral small vessel disease (196). Following oral infection with Streptococcus mutans, the bacteria bind to exposed collagen layers due to the expressed collagen-binding protein and anionic cell surface conditions, activating matrix metalloproteinase (MMP-9) and inhibiting platelet aggregation at injured blood vessels induced by collagen, potentially leading to persistent bleeding, which may be a contributing factor to cerebral microbleeds in CSVD (197). Another study evaluated nine periodontal pathogens and identified Campylobacter rectus as being associated with cerebral microbleeds (198). In conclusion, certain oral microorganisms may be implicated in systemic inflammatory responses, disruption of vascular endothelial function, and platelet abnormalities, inducing the occurrence and progression of CSVD through a variety of mechanisms.

## 9 Treatment of cerebral small vessel disease caused by pathogen infection

Despite the limited clinical evidence for the treatment of cerebrovascular small vessel disease caused by pathogen infection, it is of paramount importance and should not be overlooked. The treatment of this condition necessitates targeted interventions based on its multifaceted pathophysiological mechanisms, as the slowly evolving nature of the disease often requires long-term and sustained therapeutic measures. Beyond the conventional treatments for large artery diseases and cardioembolism, such as antiplatelet therapy, blood pressure control, lipid-lowering agents, and anticoagulation, the treatment strategies for pathogen-induced CSVD can also revolve around several core principles. The treatment of infectious CSVD requires an individualized approach tailored to the specific circumstances of each patient. Firstly, identifying the specific pathogen is fundamental to formulating an effective treatment strategy. Secondly, evaluating the severity of neuroinflammation and the integrity of the blood-brain barrier are crucial for selecting appropriate treatment modalities. Furthermore, monitoring treatment responses, adverse effects, and timely adjustments to the treatment plan are essential.

Low levels of vitamin D are associated with inflammatory responses mediated by NF-kB signaling, potentially contributing to the pathogenesis of CSVD (199). Vitamin D modulates inflammatory mediators and immune function, protects against neuronal apoptosis, and is closely linked to the gut microbiome, thereby serving as a regulator of neuroinflammation. The application of nanotechnology enables vitamin D to maximize its potential in ameliorating neuroinflammation and protecting neurons (200). Type I interferon (IFN-I) molecules produced by cells in the CNS can prevent viral infections, and early supplementation of IFN-I may be beneficial. It can also modulate neuroinflammation and potentially serve as a new therapy for cognitive decline and long-haul COVID. However, it is important to note that IFN-I may be a double-edged sword, requiring careful timing of administration and further experiments to assess its safety and efficacy (201). As anti-inflammatory and antibacterial immunomodulators, itaconate and mesaconate can ameliorate brain inflammation induced by lipopolysaccharide (LPS), a common antigen on the surface of Gram-negative bacteria, by downregulating key pro-inflammatory cytokines and reversing synaptic plasticity impairment, representing a highly promising therapeutic strategy (202). Similarly, for LPS-induced neuroinflammation, the A1 adenosine receptor (A1AR) agonist N6-2-chloro-6-fluorobenzylamino)-2-pyrimidinyl)ethylamino) ethyladenosine (CHA) induces hypothermia by activating thermoregulatory circuits, thereby preventing blood-brain barrier disruption and mitigating the adverse effects of this neuroinflammation. his also suggests that hypothermia may have therapeutic potential for CNS infectious diseases (203). Delta-9-tetrahydrocannabinol can also reduce neuroinflammation and oxidative stress, and improve dysregulation of the microbiome-gut-brain axis (MGBA), potentially offering a novel therapeutic paradigm, particularly for HIV patients (204). For individuals infected with Zika virus, 1-methyl-D-tryptophan (1-MT) inhibitors may exert neuroprotective effects by blocking the Indoleamine-2,3-dioxygenase (IDO-1) enzyme, although they cannot completely prevent inflammation, they can reduce brain damage and cell death. Future studies could investigate whether 1-MT inhibitors can be combined with antiviral drugs for the treatment of ZIKV infection (205). In a rat model of Porphyromonas gingivalis infection, researchers observed that treatment with alantolactone suppressed neuroinflammation, ameliorated oxidative stress, and mitigated cognitive impairment. These findings suggest that alantolactone may represent a novel therapeutic strategy (206). Under conditions of blood-brain barrier (BBB) compromise, antioxidants and VEGF antibodies have been shown in experiments to enhance BBB function, while regulators of cGMP (such as dipyridamole) and cAMP (such as cilostazol and pentoxifylline) can improve BBB integrity. However, research on the efficacy of these interventions during active pathogen infection remains limited (207).

Although these treatment strategies are theoretically feasible, their clinical application still faces many challenges, including the lack of direct clinical evidence, large individual differences, and difficulties in evaluating treatment effects. Therefore, future research is needed to further clarify the specific association between pathogen infection and cerebral small vessel disease, understand its pathological mechanism, develop more effective diagnostic and treatment strategies, and conduct more clinical trials to verify the efficacy and safety of these treatment strategies. With the deeper understanding of infectious CSVD and the progress of treatment technology, we have reason to believe that the treatment of cerebral small vessel disease induced by pathogen infection will be more accurate and effective, which will bring better prognosis and quality of life for patients.

## 10 Conclusion

As global life expectancy continues to rise, the prevalence of cerebral small vessel disease in an aging population is pronounced, increasing the risk of dementia, stroke, or mortality, and imposing a significant societal burden. The urgency for further research into CSVD is evident; however, it would be remiss to consider CSVD merely as a miniature version of ischemic stroke. Its pathophysiology encompasses multitude mechanisms, including immunosenescence and genetics. Although the exact pathogenesis of CSVD remains unclear, inflammation has emerged as a focal point of research, with persistent inflammatory responses thought to potentially facilitate the onset and progression of CSVD. Infections, as a pivotal trigger of inflammation, are closely intertwined with human daily life. The chronic inflammatory response following infection, often subtle or asymptomatic, is easily overlooked, leading to an underestimation of its long-term impact on the human body. Consequently, investigating the role of infections in CSVD holds considerable potential and clinical significance. We have summarized the latest findings on infection-induced CSVD and proposed various potential pathological mechanisms by which different pathogens may lead to CSVD, with the aim of providing a basis for future disease prevention and treatment. The complex interplay between infection-induced inflammation and CSVD is not yet fully understood, necessitating further exploration to facilitate the development of effective therapeutic interventions for CSVD.

## References

[1] Pantoni L. Cerebral small vessel disease: from pathogenesis and clinical characteristics to therapeutic challenges. Lancet Neurol. (2010) 9:689–701. doi: 10.1016/s1474-4422(10)70104-6 20610345

[2] Li Q Yang Y Reis C Tao T Li W Li X. Cerebral small vessel disease. Cell Transplant. (2018) 27:1711–22. doi: 10.1177/0963689718795148 PMC6300773 30251566

[3] Markus HS de Leeuw FE. Cerebral small vessel disease: recent advances and future directions. Int J Stroke. (2023) 18:4–14. doi: 10.1177/17474930221144911 36575578 PMC9806465

[4] Wardlaw JM Smith EE Biessels GJ Cordonnier C Fazekas F Frayne R. Neuroimaging standards for research into small vessel disease and its contribution to ageing and neurodegeneration. Lancet Neurol. (2013) 12:822–38. doi: 10.1016/s1474-4422(13)70124-8 PMC3714437 23867200

[5] Cannistraro RJ Badi M Eidelman BH Dickson DW Middlebrooks EH Meschia JF. Cns small vessel disease: A clinical review. Neurology. (2019) 92:1146–56. doi: 10.1212/wnl.0000000000007654 PMC6598791 31142635

[6] Staals J Makin SD Doubal FN Dennis MS Wardlaw JM. Stroke subtype, vascular risk factors, and total mri brain small-vessel disease burden. Neurology. (2014) 83:1228–34. doi: 10.1212/wnl.0000000000000837 PMC4180484 25165388

[7] Liu J Rutten-Jacobs L Liu M Markus HS Traylor M. Causal impact of type 2 diabetes mellitus on cerebral small vessel disease: A mendelian randomization analysis. Stroke. (2018) 49:1325–31. doi: 10.1161/strokeaha.117.020536 PMC5976219 29686024

[8] Tran VTA Lee LP Cho H. Neuroinflammation in neurodegeneration via microbial infections. Front Immunol. (2022) 13:907804. doi: 10.3389/fimmu.2022.907804 36052093 PMC9425114

[9] Jiang L Cai X Yao D Jing J Mei L Yang Y. Association of inflammatory markers with cerebral small vessel disease in community-based population. J Neuroinflammation. (2022) 19:106. doi: 10.1186/s12974-022-02468-0 35513834 PMC9072153

[10] Rodríguez AM Rodríguez J Giambartolomei GH. Microglia at the crossroads of pathogen-induced neuroinflammation. ASN Neuro. (2022) 14:17590914221104566. doi: 10.1177/17590914221104566 35635133 PMC9158411

[11] Li L Acioglu C Heary RF Elkabes S. Role of astroglial toll-like receptors (Tlrs) in central nervous system infections, injury and neurodegenerative diseases. Brain Behav Immun. (2021) 91:740–55. doi: 10.1016/j.bbi.2020.10.007 PMC7543714 33039660

[12] DiSabato DJ Quan N Godbout JP. Neuroinflammation: the devil is in the details. J Neurochem. (2016) 139 Suppl 2:136–53. doi: 10.1111/jnc.13607 PMC5025335 26990767

[13] Abbott NJ Rönnbäck L Hansson E. Astrocyte-endothelial interactions at the blood-brain barrier. Nat Rev Neurosci. (2006) 7:41–53. doi: 10.1038/nrn1824 16371949

[14] Hanke ML Kielian T. Toll-like receptors in health and disease in the brain: mechanisms and therapeutic potential. Clin Sci (Lond). (2011) 121:367–87. doi: 10.1042/cs20110164 PMC4231819 21745188

[15] Patabendige A Janigro D. The role of the blood-brain barrier during neurological disease and infection. Biochem Soc Trans. (2023) 51:613–26. doi: 10.1042/bst20220830 PMC10212550 36929707

[16] Jian B Hu M Cai W Zhang B Lu Z. Update of immunosenescence in cerebral small vessel disease. Front Immunol. (2020) 11:585655. doi: 10.3389/fimmu.2020.585655 33362768 PMC7756147

[17] Pavlov VA Chavan SS Tracey KJ. Molecular and functional neuroscience in immunity. Annu Rev Immunol. (2018) 36:783–812. doi: 10.1146/annurev-immunol-042617-053158 29677475 PMC6057146

[18] Pellegrini L Albecka A Mallery DL Kellner MJ Paul D Carter AP. Sars-cov-2 infects the brain choroid plexus and disrupts the blood-csf barrier in human brain organoids. Cell Stem Cell. (2020) 27:951–61.e5. doi: 10.1016/j.stem.2020.10.001 33113348 PMC7553118

[19] Chen R Wang K Yu J Howard D French L Chen Z. The spatial and cell-type distribution of sars-cov-2 receptor ace2 in the human and mouse brains. Front Neurol. (2020) 11:573095. doi: 10.3389/fneur.2020.573095 33551947 PMC7855591

[20] Wu Y Xu X Chen Z Duan J Hashimoto K Yang L. Nervous system involvement after infection with covid-19 and other coronaviruses. Brain Behav Immun. (2020) 87:18–22. doi: 10.1016/j.bbi.2020.03.031 32240762 PMC7146689

[21] Bonaventura A Vecchié A Dagna L Martinod K Dixon DL Van Tassell BW. Endothelial dysfunction and immunothrombosis as key pathogenic mechanisms in covid-19. Nat Rev Immunol. (2021) 21:319–29. doi: 10.1038/s41577-021-00536-9 PMC8023349 33824483

[22] Otifi HM Adiga BK. Endothelial dysfunction in covid-19 infection. Am J Med Sci. (2022) 363:281–7. doi: 10.1016/j.amjms.2021.12.010 PMC8802031 35093394

[23] DeOre BJ Tran KA Andrews AM Ramirez SH Galie PA. Sars-cov-2 spike protein disrupts blood-brain barrier integrity via rhoa activation. J Neuroimmune Pharmacol. (2021) 16:722–8. doi: 10.1007/s11481-021-10029-0 PMC8536479 34687399

[24] Rhea EM Logsdon AF Hansen KM Williams LM Reed MJ Baumann KK. The S1 protein of sars-cov-2 crosses the blood-brain barrier in mice. Nat Neurosci. (2021) 24:368–78. doi: 10.1038/s41593-020-00771-8 PMC8793077 33328624

[25] Perico L Benigni A Remuzzi G. Sars-cov-2 and the spike protein in endotheliopathy. Trends Microbiol. (2024) 32:53–67. doi: 10.1016/j.tim.2023.06.004 37393180 PMC10258582

[26] Boluda S Mokhtari K Mégarbane B Annane D Mathon B Cao A. Golgi localization of sars-cov-2 spike protein and interaction with furin in cerebral covid-19 microangiopathy: A clue to the central nervous system involvement? Free Neuropathol. (2023) 4. doi: 10.17879/freeneuropathology-2023-4584 PMC10240951 37283933

[27] Fontes-Dantas FL Fernandes GG Gutman EG De Lima EV Antonio LS Hammerle MB. Sars-cov-2 spike protein induces tlr4-mediated long-term cognitive dysfunction recapitulating post-covid-19 syndrome in mice. Cell Rep. (2023) 42:112189. doi: 10.1016/j.celrep.2023.112189 36857178 PMC9935273

[28] Garcia-Larragoiti N Cano-Mendez A Jimenez-Vega Y Trujillo M Guzman-Cancino P Ambriz-Murillo Y. Inflammatory and prothrombotic biomarkers contribute to the persistence of sequelae in recovered covid-19 patients. Int J Mol Sci. (2023) 24. doi: 10.3390/ijms242417468 PMC10744310 38139298

[29] Hamzeh-Cognasse H Mansour A Reizine F Mismetti P Gouin-Thibault I Cognasse F. Platelet-derived scd40l: specific inflammatory marker for early-stage severe acute respiratory syndrome coronavirus 2 infection. Virol J. (2021) 18:211. doi: 10.1186/s12985-021-01680-3 34715884 PMC8554745

[30] Wu Y Wang M Yin H Ming S Li X Jiang G. Trem-2 is a sensor and activator of T cell response in sars-cov-2 infection. Sci Adv. (2021) 7:eabi6802. doi: 10.1126/sciadv.abi6802 34878838 PMC8654301

[31] Forcados GE Muhammad A Oladipo OO Makama S Meseko CA. Metabolic implications of oxidative stress and inflammatory process in sars-cov-2 pathogenesis: therapeutic potential of natural antioxidants. Front Cell Infect Microbiol. (2021) 11:654813. doi: 10.3389/fcimb.2021.654813 34123871 PMC8188981

[32] Hameedi MA TP E Garvin MR Mathews II Amos BK Demerdash O. Structural and functional characterization of nemo cleavage by sars-cov-2 3clpro. Nat Commun. (2022) 13:5285. doi: 10.1038/s41467-022-32922-9 36075915 PMC9453703

[33] Wenzel J Lampe J Müller-Fielitz H Schuster R Zille M Müller K. The sars-cov-2 main protease M(Pro) causes microvascular brain pathology by cleaving nemo in brain endothelial cells. Nat Neurosci. (2021) 24:1522–33. doi: 10.1038/s41593-021-00926-1 PMC8553622 34675436

[34] Owens CD Bonin Pinto C Detwiler S Olay L Pinaffi-Langley A Mukli P. Neurovasculaw3r coupling impairment as a mechanism for cognitive deficits in covid-19. Brain Commun. (2024) 6:fcae080. doi: 10.1093/braincomms/fcae080 38495306 PMC10943572

[35] Che Mohd Nassir CMN Hashim S Wong KK Abdul Halim S Idris NS Jayabalan N. Covid-19 infection and circulating microparticles-reviewing evidence as microthrombogenic risk factor for cerebral small vessel disease. Mol Neurobiol. (2021) 58:4188–215. doi: 10.1007/s12035-021-02457-z PMC8235918 34176095

[36] Soontornniyomkij V Umlauf A Chung SA Cochran ML Soontornniyomkij B Gouaux B. Hiv protease inhibitor exposure predicts cerebral small vessel disease. Aids. (2014) 28:1297–306. doi: 10.1097/qad.0000000000000262 PMC4071161 24637542

[37] Price RW Peterson J Fuchs D Angel TE Zetterberg H Hagberg L. Approach to cerebrospinal fluid (Csf) biomarker discovery and evaluation in hiv infection. J Neuroimmune Pharmacol. (2013) 8:1147–58. doi: 10.1007/s11481-013-9491-3 PMC3889225 23943280

[38] Burdo TH Lentz MR Autissier P Krishnan A Halpern E Letendre S. Soluble cd163 made by monocyte/macrophages is a novel marker of hiv activity in early and chronic infection prior to and after anti-retroviral therapy. J Infect Dis. (2011) 204:154–63. doi: 10.1093/infdis/jir214 PMC3105035 21628670

[39] Trøseid M Nowak P Nyström J Lindkvist A Abdurahman S Sönnerborg A. Elevated plasma levels of lipopolysaccharide and high mobility group box-1 protein are associated with high viral load in hiv-1 infection: reduction by 2-year antiretroviral therapy. Aids. (2010) 24:1733–7. doi: 10.1097/QAD.0b013e32833b254d 20502315

[40] Fletcher NF Wilson GK Murray J Hu K Lewis A Reynolds GM. Hepatitis C virus infects the endothelial cells of the blood-brain barrier. Gastroenterology. (2012) 142:634–43.e6. doi: 10.1053/j.gastro.2011.11.028 22138189 PMC3801216

[41] Yarlott L Heald E Forton D. Hepatitis C virus infection, and neurological and psychiatric disorders - a review. J Adv Res. (2017) 8:139–48. doi: 10.1016/j.jare.2016.09.005 PMC5272938 28149649

[42] Liu Y Chen L Zou Z Zhu B Hu Z Zeng P. Hepatitis C virus infection induces elevation of cxcl10 in human brain microvascular endothelial cells. J Med Virol. (2016) 88:1596–603. doi: 10.1002/jmv.24504 26895737

[43] Ayala-Nunez NV Follain G Delalande F Hirschler A Partiot E Hale GL. Zika virus enhances monocyte adhesion and transmigration favoring viral dissemination to neural cells. Nat Commun. (2019) 10:4430. doi: 10.1038/s41467-019-12408-x 31562326 PMC6764950

[44] Bhardwaj U Singh SK. Zika virus ns1 suppresses ve-cadherin via hsa-mir-29b-3p/dnmt3b/mmp-9 pathway in human brain microvascular endothelial cells. Cell Signal. (2023) 106:110659. doi: 10.1016/j.cellsig.2023.110659 36948479

[45] Zhou J Chi X Cheng M Huang X Liu X Fan J. Zika virus degrades the Ω-3 fatty acid transporter mfsd2a in brain microvascular endothelial cells and impairs lipid homeostasis. Sci Adv. (2019) 5:eaax7142. doi: 10.1126/sciadv.aax7142 31681849 PMC6810275

[46] Lu S Wang J He Z He S Zheng K Xu M. Treponema pallidum tp0751 alters the expression of tight junction proteins by promoting bend3 cell apoptosis and il-6 secretion. Int J Med Microbiol. (2022) 312:151553. doi: 10.1016/j.ijmm.2022.151553 35358795

[47] Xu DM Cai SN Li R Wu Y Liu SA Lun WH. Elevation of cerebrospinal fluid light and heavy neurofilament levels in symptomatic neurosyphilis. Sex Transm Dis. (2020) 47:634–8. doi: 10.1097/olq.0000000000001236 PMC7447120 32649582

[48] Fongang B Satizabal C Kautz TF Wadop YN Muhammad JAS Vasquez E. Cerebral small vessel disease burden is associated with decreased abundance of gut barnesiella intestinihominis bacterium in the framingham heart study. Sci Rep. (2023) 13:13622. doi: 10.1038/s41598-023-40872-5 37604954 PMC10442369

[49] Shi Y Zhao E Li L Zhao S Mao H Deng J. Alteration and clinical potential in gut microbiota in patients with cerebral small vessel disease. Front Cell Infect Microbiol. (2023) 13:1231541. doi: 10.3389/fcimb.2023.1231541 37496806 PMC10366612

[50] Cai W Chen X Men X Ruan H Hu M Liu S. Gut microbiota from patients with arteriosclerotic csvd induces higher il-17a production in neutrophils via activating rorγt. Sci Adv. (2021) 7. doi: 10.1126/sciadv.abe4827 PMC10964962 33523954

[51] Liu S Men X Guo Y Cai W Wu R Gao R. Gut microbes exacerbate systemic inflammation and behavior disorders in neurologic disease cadasil. Microbiome. (2023) 11:202. doi: 10.1186/s40168-023-01638-3 37684694 PMC10486110

[52] Walter C Zahlten J Schmeck B Schaudinn C Hippenstiel S Frisch E. Porphyromonas gingivalis strain-dependent activation of human endothelial cells. Infect Immun. (2004) 72:5910–8. doi: 10.1128/iai.72.10.5910-5918.2004 PMC517532 15385493

[53] Aarabi G Thomalla G Heydecke G Seedorf U. Chronic oral infection: an emerging risk factor of cerebral small vessel disease. Dis. (2019) 25:710–9. doi: 10.1111/odi.12912 29878487

[54] Lourbakos A Yuan YP Jenkins AL Travis J Andrade-Gordon P Santulli R. Activation of protease-activated receptors by gingipains from porphyromonas gingivalis leads to platelet aggregation: A new trait in microbial pathogenicity. Blood. (2001) 97:3790–7. doi: 10.1182/blood.v97.12.3790 11389018

[55] Hosoki S Saito S Tonomura S Ishiyama H Yoshimoto T Ikeda S. Oral carriage of streptococcus mutans harboring the cnm gene relates to an increased incidence of cerebral microbleeds. Stroke. (2020) 51:3632–9. doi: 10.1161/strokeaha.120.029607 PMC7678651 33148146

[56] Hu B Guo H Zhou P Shi ZL. Characteristics of sars-cov-2 and covid-19. Nat Rev Microbiol. (2021) 19:141–54. doi: 10.1038/s41579-020-00459-7 PMC7537588 33024307

[57] Shereen MA Khan S Kazmi A Bashir N Siddique R. Covid-19 infection: origin, transmission, and characteristics of human coronaviruses. J Adv Res. (2020) 24:91–8. doi: 10.1016/j.jare.2020.03.005 PMC7113610 32257431

[58] Meselson M. Droplets and aerosols in the transmission of sars-cov-2. N Engl J Med. (2020) 382:2063. doi: 10.1056/NEJMc2009324 32294374 PMC7179963

[59] Shah MD Sumeh AS Sheraz M Kavitha MS Venmathi Maran BA Rodrigues KF. A mini-review on the impact of covid 19 on vital organs. BioMed Pharmacother. (2021) 143:112158. doi: 10.1016/j.biopha.2021.112158 34507116 PMC8416601

[60] Hensley MK Markantone D Prescott HC. Neurologic manifestations and complications of covid-19. Annu Rev Med. (2022) 73:113–27. doi: 10.1146/annurev-med-042320-010427 34416121

[61] Singh D Yi SV. On the origin and evolution of sars-cov-2. Exp Mol Med. (2021) 53:537–47. doi: 10.1038/s12276-021-00604-z PMC8050477 33864026

[62] Owens CD Pinto CB Detwiler S Mukli P Peterfi A Szarvas Z. Cerebral small vessel disease pathology in covid-19 patients: A systematic review. Ageing Res Rev. (2023) 88:101962. doi: 10.1016/j.arr.2023.101962 37224885 PMC10202464

[63] Iadecola C Anrather J Kamel H. Effects of covid-19 on the nervous system. Cell. (2020) 183:16–27.e1. doi: 10.1016/j.cell.2020.08.028 32882182 PMC7437501

[64] Gusev E Sarapultsev A Solomatina L Chereshnev V. Sars-cov-2-specific immune response and the pathogenesis of covid-19. Int J Mol Sci. (2022) 23. doi: 10.3390/ijms23031716 PMC8835786 35163638

[65] Wang Y Perlman S. Covid-19: inflammatory profile. Annu Rev Med. (2022) 73:65–80. doi: 10.1146/annurev-med-042220-012417 34437814

[66] Ali N. Elevated level of C-reactive protein may be an early marker to predict risk for severity of covid-19. J Med Virol. (2020) 92:2409–11. doi: 10.1002/jmv.26097 PMC7301027 32516845

[67] Yao Y Cao J Wang Q Shi Q Liu K Luo Z. D-dimer as a biomarker for disease severity and mortality in covid-19 patients: A case control study. J Intensive Care. (2020) 8:49. doi: 10.1186/s40560-020-00466-z 32665858 PMC7348129

[68] Mandal S Barnett J Brill SE Brown JS Denneny EK Hare SS. ‘Long-covid’: A cross-sectional study of persisting symptoms, biomarker and imaging abnormalities following hospitalisation for covid-19. Thorax. (2021) 76:396–8. doi: 10.1136/thoraxjnl-2020-215818 PMC7615158 33172844

[69] Leatherdale A Stukas S Lei V West HE Campbell CJ Hoiland RL. Persistently elevated complement alternative pathway biomarkers in covid-19 correlate with hypoxemia and predict in-hospital mortality. Med Microbiol Immunol. (2022) 211:37–48. doi: 10.1007/s00430-021-00725-2 35034207 PMC8761108

[70] Ponti G Maccaferri M Ruini C Tomasi A Ozben T. Biomarkers associated with covid-19 disease progression. Crit Rev Clin Lab Sci. (2020) 57:389–99. doi: 10.1080/10408363.2020.1770685 PMC7284147 32503382

[71] Shoamanesh A Preis SR Beiser AS Vasan RS Benjamin EJ Kase CS. Inflammatory biomarkers, cerebral microbleeds, and small vessel disease: framingham heart study. Neurology. (2015) 84:825–32. doi: 10.1212/wnl.0000000000001279 PMC4345647 25632086

[72] Wan S Dandu C Han G Guo Y Ding Y Song H. Plasma inflammatory biomarkers in cerebral small vessel disease: A review. CNS Neurosci Ther. (2023) 29:498–515. doi: 10.1111/cns.14047 36478511 PMC9873530

[73] Gorog DA Storey RF Gurbel PA Tantry US Berger JS Chan MY. Current and novel biomarkers of thrombotic risk in covid-19: A consensus statement from the international covid-19 thrombosis biomarkers colloquium. Nat Rev Cardiol. (2022) 19:475–95. doi: 10.1038/s41569-021-00665-7 PMC8757397 35027697

[74] Tang T Cheng X Truong B Sun L Yang X Wang H. Molecular basis and therapeutic implications of cd40/cd40l immune checkpoint. Pharmacol Ther. (2021) 219:107709. doi: 10.1016/j.pharmthera.2020.107709 33091428 PMC7886970

[75] Zaid Y Puhm F Allaeys I Naya A Oudghiri M Khalki L. Platelets can associate with sars-cov-2 rna and are hyperactivated in covid-19. Circ Res. (2020) 127:1404–18. doi: 10.1161/circresaha.120.317703 PMC7641188 32938299

[76] Savarraj J Park ES Colpo GD Hinds SN Morales D Ahnstedt H. Brain injury, endothelial injury and inflammatory markers are elevated and express sex-specific alterations after covid-19. J Neuroinflammation. (2021) 18:277. doi: 10.1186/s12974-021-02323-8 34838058 PMC8627162

[77] Chen K Huang J Gong W Zhang L Yu P Wang JM. Cd40/cd40l dyad in the inflammatory and immune responses in the central nervous system. Cell Mol Immunol. (2006) 3:163–9. 16893496

[78] Bhat SA Goel R Shukla R Hanif K. Platelet cd40l induces activation of astrocytes and microglia in hypertension. Brain Behav Immun. (2017) 59:173–89. doi: 10.1016/j.bbi.2016.09.021 27658543

[79] Masuda H Mori M Uchida T Uzawa A Ohtani R Kuwabara S. Soluble cd40 ligand contributes to blood-brain barrier breakdown and central nervous system inflammation in multiple sclerosis and neuromyelitis optica spectrum disorder. J Neuroimmunol. (2017) 305:102–7. doi: 10.1016/j.jneuroim.2017.01.024 28284329

[80] Zhong X Wang H Ye Z Qiu W Lu Z Li R. Serum concentration of cd40l is elevated in inflammatory demyelinating diseases. J Neuroimmunol. (2016) 299:66–9. doi: 10.1016/j.jneuroim.2016.08.015 27725124

[81] Yu S Liu YP Liu YH Jiao SS Liu L Wang YJ. Diagnostic utility of vegf and soluble cd40l levels in serum of alzheimer’s patients. Clin Chim Acta. (2016) 453:154–9. doi: 10.1016/j.cca.2015.12.018 26706786

[82] Staszewski J Piusińska-Macoch R Brodacki B Skrobowska E Stępień A. Il-6, pf-4, scd40 L, and homocysteine are associated with the radiological progression of cerebral small-vessel disease: A 2-year follow-up study. Clin Interv Aging. (2018) 13:1135–41. doi: 10.2147/cia.S166773 PMC6016008 29950823

[83] Fan R Cheng Z Huang Z Yang Y Sun N Hu B. Trem-1, trem-2 and their association with disease severity in patients with covid-19. Ann Med. (2023) 55:2269558. doi: 10.1080/07853890.2023.2269558 37848000 PMC10583614

[84] Zheng H Jia L Liu CC Rong Z Zhong L Yang L. Trem2 promotes microglial survival by activating wnt/B-catenin pathway. J Neurosci. (2017) 37:1772–84. doi: 10.1523/jneurosci.2459-16.2017 PMC5320608 28077724

[85] Ferri E Rossi PD Geraci A Ciccaone S Cesari M Arosio B. The strem2 concentrations in the blood: A marker of neurodegeneration? Front Mol Biosci. (2020) 7:627931. doi: 10.3389/fmolb.2020.627931 33768114 PMC7985346

[86] Wu C Ma YH Hu H Zhao B Tan L. Soluble trem2, alzheimer’s disease pathology, and risk for progression of cerebral small vessel disease: A longitudinal study. J Alzheimers Dis. (2023) 92:311–22. doi: 10.3233/jad-220731 36744335

[87] Chernyak BV Popova EN Prikhodko AS Grebenchikov OA Zinovkina LA Zinovkin RA. Covid-19 and oxidative stress. Biochem (Mosc). (2020) 85:1543–53. doi: 10.1134/s0006297920120068 PMC7768996 33705292

[88] Choi DH Lee KH Kim JH Seo JH Kim HY Shin CY. Nadph oxidase 1, a novel molecular source of ros in hippocampal neuronal death in vascular dementia. Antioxid Redox Signal. (2014) 21:533–50. doi: 10.1089/ars.2012.5129 PMC4086030 24294978

[89] Kahles T Luedike P Endres M Galla HJ Steinmetz H Busse R. Nadph oxidase plays a central role in blood-brain barrier damage in experimental stroke. Stroke. (2007) 38:3000–6. doi: 10.1161/strokeaha.107.489765 17916764

[90] De Silva TM Brait VH Drummond GR Sobey CG Miller AA. Nox2 oxidase activity accounts for the oxidative stress and vasomotor dysfunction in mouse cerebral arteries following ischemic stroke. PloS One. (2011) 6:e28393. doi: 10.1371/journal.pone.0028393 22164282 PMC3229592

[91] Kleinschnitz C Grund H Wingler K Armitage ME Jones E Mittal M. Post-stroke inhibition of induced nadph oxidase type 4 prevents oxidative stress and neurodegeneration. PloS Biol. (2010) 8. doi: 10.1371/journal.pbio.1000479 PMC2943442 20877715

[92] De Silva TM Miller AA. Cerebral small vessel disease: targeting oxidative stress as a novel therapeutic strategy? Front Pharmacol. (2016) 7:61. doi: 10.3389/fphar.2016.00061 27014073 PMC4794483

[93] Qiao J Li YS Zeng R Liu FL Luo RH Huang C. Sars-cov-2 M(Pro) inhibitors with antiviral activity in a transgenic mouse model. Science. (2021) 371:1374–8. doi: 10.1126/science.abf1611 PMC8099175 33602867

[94] Jiang Y Müller K Khan MA Assmann JC Lampe J Kilau K. Cerebral angiogenesis ameliorates pathological disorders in nemo-deficient mice with small-vessel disease. J Cereb Blood Flow Metab. (2021) 41:219–35. doi: 10.1177/0271678x20910522 PMC8369998 32151223

[95] Schaeffer S Iadecola C. Revisiting the neurovascular unit. Nat Neurosci. (2021) 24:1198–209. doi: 10.1038/s41593-021-00904-7 PMC9462551 34354283

[96] Kaplan L Chow BW Gu C. Neuronal regulation of the blood-brain barrier and neurovascular coupling. Nat Rev Neurosci. (2020) 21:416–32. doi: 10.1038/s41583-020-0322-2 PMC8934575 32636528

[97] Phillips AA Chan FH Zheng MM Krassioukov AV Ainslie PN. Neurovascular coupling in humans: physiology, methodological advances and clinical implications. J Cereb Blood Flow Metab. (2016) 36:647–64. doi: 10.1177/0271678x15617954 PMC4821024 26661243

[98] Yang S Webb AJS. Associations between neurovascular coupling and cerebral small vessel disease: A systematic review and meta-analysis. Eur Stroke J. (2023) 8:895–903. doi: 10.1177/23969873231196981 37697725 PMC10683738

[99] El-Gamal H Parray AS Mir FA Shuaib A Agouni A. Circulating microparticles as biomarkers of stroke: A focus on the value of endothelial- and platelet-derived microparticles. J Cell Physiol. (2019) 234:16739–54. doi: 10.1002/jcp.28499 30912147

[100] Delabranche X Berger A Boisramé-Helms J Meziani F. Microparticles and infectious diseases. Med Mal Infect. (2012) 42:335–43. doi: 10.1016/j.medmal.2012.05.011 22766273

[101] Mause SF Weber C. Microparticles: protagonists of a novel communication network for intercellular information exchange. Circ Res. (2010) 107:1047–57. doi: 10.1161/circresaha.110.226456 21030722

[102] Puddu P Puddu GM Cravero E Muscari S Muscari A. The involvement of circulating microparticles in inflammation, coagulation and cardiovascular diseases. Can J Cardiol. (2010) 26:140–5. doi: 10.1016/s0828-282x(10)70371-8 PMC2886541 20386775

[103] Nassir C Ghazali MM Safri AA Jaffer U Abdullah WZ Idris NS. Elevated circulating microparticle subpopulations in incidental cerebral white matter hyperintensities: A multimodal study. Brain Sci. (2021) 11. doi: 10.3390/brainsci11020133 PMC7909442 33498429

[104] Takei Y Yamada M Saito K Kameyama Y Sugiura H Makiguchi T. Increase in circulating ace-positive endothelial microparticles during acute lung injury. Eur Respir J. (2019) 54. doi: 10.1183/13993003.01188-2018 31320458

[105] Thom SR Bhopale VM Yu K Huang W Kane MA Margolis DJ. Neutrophil microparticle production and inflammasome activation by hyperglycemia due to cytoskeletal instability. J Biol Chem. (2017) 292:18312–24. doi: 10.1074/jbc.M117.802629 PMC5672053 28972154

[106] Briggs JA Kräusslich HG. The molecular architecture of hiv. J Mol Biol. (2011) 410:491–500. doi: 10.1016/j.jmb.2011.04.021 21762795

[107] Novikova M Adams LJ Fontana J Gres AT Balasubramaniam M Winkler DC. Identification of a structural element in hiv-1 gag required for virus particle assembly and maturation. mBio. (2018) 9. doi: 10.1128/mBio.01567-18 PMC6191540 30327442

[108] Freed EO. Hiv-1 assembly, release and maturation. Nat Rev Microbiol. (2015) 13:484–96. doi: 10.1038/nrmicro3490 PMC6936268 26119571

[109] Lucas S Nelson AM. Hiv and the spectrum of human disease. J Pathol. (2015) 235:229–41. doi: 10.1002/path.4449 25251832

[110] Hemelaar J Elangovan R Yun J Dickson-Tetteh L Fleminger I Kirtley S. Global and regional molecular epidemiology of hiv-1, 1990-2015: A systematic review, global survey, and trend analysis. Lancet Infect Dis. (2019) 19:143–55. doi: 10.1016/s1473-3099(18)30647-9 30509777

[111] Su T Wit FW Caan MW Schouten J Prins M Geurtsen GJ. White matter hyperintensities in relation to cognition in hiv-infected men with sustained suppressed viral load on combination antiretroviral therapy. Aids. (2016) 30:2329–39. doi: 10.1097/qad.0000000000001133 27149087

[112] Moulignier A Viret-Vilayphon AC Lescure FX Plaisier E Salomon L Lamirel C. Microalbuminuria: A sentinel of neurocognitive impairment in hiv-infected individuals? J Neurol. (2020) 267:1368–76. doi: 10.1007/s00415-019-09674-6 PMC7184056 31980868

[113] Zayyad Z Spudich S. Neuropathogenesis of hiv: from initial neuroinvasion to hiv-associated neurocognitive disorder (Hand). Curr HIV/AIDS Rep. (2015) 12:16–24. doi: 10.1007/s11904-014-0255-3 25604237 PMC4741099

[114] Moulignier A Savatovsky J Assoumou L Lescure FX Lamirel C Godin O. Silent cerebral small-vessel disease is twice as prevalent in middle-aged individuals with well-controlled, combination antiretroviral therapy-treated human immunodeficiency virus (Hiv) than in hiv-uninfected individuals. Clin Infect Dis. (2018) 66:1762–9. doi: 10.1093/cid/cix1075 29244126

[115] Januel E Godin O Moulignier A Lescure FX Savatovsky J Lamirel C. Brief report: impact of art classes on the increasing risk of cerebral small-vessel disease in middle-aged, well-controlled, cart-treated, hiv-infected individuals. J Acquir Immune Defic Syndr. (2019) 81:547–51. doi: 10.1097/qai.0000000000002084 31107300

[116] Sanford R Strain J Dadar M Maranzano J Bonnet A Mayo NE. Hiv infection and cerebral small vessel disease are independently associated with brain atrophy and cognitive impairment. Aids. (2019) 33:1197–205. doi: 10.1097/qad.0000000000002193 PMC7924885 30870193

[117] Miyaue N Yamanishi Y Ito Y Ando R Nagai M. Csf neopterin levels are elevated in various neurological diseases and aging. J Clin Med. (2024) 13. doi: 10.3390/jcm13154542 PMC11312611 39124808

[118] Imp BM Rubin LH Tien PC Plankey MW Golub ET French AL. Monocyte activation is associated with worse cognitive performance in hiv-infected women with virologic suppression. J Infect Dis. (2017) 215:114–21. doi: 10.1093/infdis/jiw506 PMC5225255 27789726

[119] Cirillo P Pacileo M DE Rosa S Calabrò P Gargiulo A Angri V. Neopterin induces pro-atherothrombotic phenotype in human coronary endothelial cells. J Thromb Haemost. (2006) 4:2248–55. doi: 10.1111/j.1538-7836.2006.02125.x 16842491

[120] Rouhl RP Damoiseaux JG Lodder J Theunissen RO Knottnerus IL Staals J. Vascular inflammation in cerebral small vessel disease. Neurobiol Aging. (2012) 33:1800–6. doi: 10.1016/j.neurobiolaging.2011.04.008 21601314

[121] Weaver LK Hintz-Goldstein KA Pioli PA Wardwell K Qureshi N Vogel SN. Pivotal advance: activation of cell surface toll-like receptors causes shedding of the hemoglobin scavenger receptor cd163. J Leukoc Biol. (2006) 80:26–35. doi: 10.1189/jlb.1205756 16799153

[122] Panahi M Hase Y Gallart-Palau X Mitra S Watanabe A Low RC. Er stress induced immunopathology involving complement in cadasil: implications for therapeutics. Acta Neuropathol Commun. (2023) 11:76. doi: 10.1186/s40478-023-01558-1 37158955 PMC10169505

[123] Morton L Arndt P Garza AP Henneicke S Mattern H Gonzalez M. Spatio-temporal dynamics of microglia phenotype in human and murine csvd: impact of acute and chronic hypertensive states. Acta Neuropathol Commun. (2023) 11:204. doi: 10.1186/s40478-023-01672-0 38115109 PMC10729582

[124] Travers AA. Priming the nucleosome: A role for hmgb proteins? EMBO Rep. (2003) 4:131–6. doi: 10.1038/sj.embor.embor741 PMC1315838 12612600

[125] Scaffidi P Misteli T Bianchi ME. Release of chromatin protein hmgb1 by necrotic cells triggers inflammation. Nature. (2002) 418:191–5. doi: 10.1038/nature00858 12110890

[126] Xue J Suarez JS Minaai M Li S Gaudino G Pass HI. Hmgb1 as a therapeutic target in disease. J Cell Physiol. (2021) 236:3406–19. doi: 10.1002/jcp.30125 PMC8104204 33107103

[127] Wang M Liu J Wang F Li Q Zhang J Ji S. The correlation between the severity of cerebral microbleeds and serum hmgb1 levels and cognitive impairment in patients with cerebral small vessel disease. Front Aging Neurosci. (2023) 15:1221548. doi: 10.3389/fnagi.2023.1221548 37424630 PMC10325658

[128] Suzuki T Aizaki H Murakami K Shoji I Wakita T. Molecular biology of hepatitis C virus. J Gastroenterol. (2007) 42:411–23. doi: 10.1007/s00535-007-2030-3 17671755

[129] Tellinghuisen TL Rice CM. Interaction between hepatitis C virus proteins and host cell factors. Curr Opin Microbiol. (2002) 5:419–27. doi: 10.1016/s1369-5274(02)00341-7 12160863

[130] Chevaliez S Pawlotsky JM. Hepatitis C virus: virology, diagnosis and management of antiviral therapy. World J Gastroenterol. (2007) 13:2461–6. doi: 10.3748/wjg.v13.i17.2461 PMC4146765 17552030

[131] Bartenschlager R Cosset FL Lohmann V. Hepatitis C virus replication cycle. J Hepatol. (2010) 53:583–5. doi: 10.1016/j.jhep.2010.04.015 20579761

[132] Preciado MV Valva P Escobar-Gutierrez A Rahal P Ruiz-Tovar K Yamasaki L. Hepatitis C virus molecular evolution: transmission, disease progression and antiviral therapy. World J Gastroenterol. (2014) 20:15992–6013. doi: 10.3748/wjg.v20.i43.15992 PMC4239486 25473152

[133] Mazzaro C Quartuccio L Adinolfi LE Roccatello D Pozzato G Nevola R. A review on extrahepatic manifestations of chronic hepatitis C virus infection and the impact of direct-acting antiviral therapy. Viruses. (2021) 13. doi: 10.3390/v13112249 PMC8619859 34835054

[134] Negro F Esmat G. Extrahepatic manifestations in hepatitis C virus infection. J Adv Res. (2017) 8:85–7. doi: 10.1016/j.jare.2016.08.004 PMC5272942 28149644

[135] Cacoub P Comarmond C Domont F Savey L Desbois AC Saadoun D. Extrahepatic manifestations of chronic hepatitis C virus infection. Ther Adv Infect Dis. (2016) 3:3–14. doi: 10.1177/2049936115585942 26862398 PMC4735500

[136] Monaco S Ferrari S Gajofatto A Zanusso G Mariotto S. Hcv-related nervous system disorders. Clin Dev Immunol. (2012) 2012:236148. doi: 10.1155/2012/236148 22899946 PMC3414089

[137] Boddi M Abbate R Chellini B Giusti B Giannini C Pratesi G. Hepatitis C virus rna localization in human carotid plaques. J Clin Virol. (2010) 47:72–5. doi: 10.1016/j.jcv.2009.10.005 19896417

[138] Lee MH Yang HI Wang CH Jen CL Yeh SH Liu CJ. Hepatitis C virus infection and increased risk of cerebrovascular disease. Stroke. (2010) 41:2894–900. doi: 10.1161/strokeaha.110.598136 20966408

[139] Morgello S Estanislao L Ryan E Gerits P Simpson D Verma S. Effects of hepatic function and hepatitis C virus on the nervous system assessment of advanced-stage hiv-infected individuals. Aids. (2005) 19 Suppl 3:S116–22. doi: 10.1097/01.aids.0000192079.49185.f9 16251806

[140] Jernigan TL Archibald SL Fennema-Notestine C Taylor MJ Theilmann RJ Julaton MD. Clinical factors related to brain structure in hiv: the charter study. J Neurovirol. (2011) 17:248–57. doi: 10.1007/s13365-011-0032-7 PMC3702821 21544705

[141] Morgello S Murray J van der Elst S Byrd D. Hcv, but not hiv, is a risk factor for cerebral small vessel disease. Neurol Neuroimmunol Neuroinflamm. (2014) 1:e27. doi: 10.1212/nxi.0000000000000027 25340079 PMC4204233

[142] White MK Wollebo HS David Beckham J Tyler KL Khalili K. Zika virus: an emergent neuropathological agent. Ann Neurol. (2016) 80:479–89. doi: 10.1002/ana.24748 PMC5086418 27464346

[143] Musso D Gubler DJ. Zika virus. Clin Microbiol Rev. (2016) 29:487–524. doi: 10.1128/cmr.00072-15 27029595 PMC4861986

[144] Christian KM Song H Ming GL. Pathophysiology and mechanisms of zika virus infection in the nervous system. Annu Rev Neurosci. (2019) 42:249–69. doi: 10.1146/annurev-neuro-080317-062231 PMC7523638 31283901

[145] Pielnaa P Al-Saadawe M Saro A Dama MF Zhou M Huang Y. Zika virus-spread, epidemiology, genome, transmission cycle, clinical manifestation, associated challenges, vaccine and antiviral drug development. Virology. (2020) 543:34–42. doi: 10.1016/j.virol.2020.01.015 32056845

[146] Munoz-Jordan JL. Diagnosis of zika virus infections: challenges and opportunities. J Infect Dis. (2017) 216:S951–s6. doi: 10.1093/infdis/jix502 PMC5853979 29267922

[147] Pierson TC Diamond MS. The emergence of zika virus and its new clinical syndromes. Nature. (2018) 560:573–81. doi: 10.1038/s41586-018-0446-y 30158602

[148] Rivera-Correa J de Siqueira IC Mota S do Rosário MS Pereira de Jesus PA Alcantara LCJ. Anti-ganglioside antibodies in patients with zika virus infection-associated guillain-barré Syndrome in Brazil. PloS Negl Trop Dis. (2019) 13:e0007695. doi: 10.1371/journal.pntd.0007695 31527907 PMC6764688

[149] Lynch RM Mantus G Encinales L Pacheco N Li G Porras A. Augmented zika and dengue neutralizing antibodies are associated with guillain-barré Syndrome. J Infect Dis. (2019) 219:26–30. doi: 10.1093/infdis/jiy466 30113672 PMC6284544

[150] Mladinich MC Schwedes J Mackow ER. Zika virus persistently infects and is basolaterally released from primary human brain microvascular endothelial cells. mBio. (2017) 8. doi: 10.1128/mBio.00952-17 PMC5513708 28698279

[151] Clé M Desmetz C Barthelemy J Martin MF Constant O Maarifi G. Zika virus infection promotes local inflammation, cell adhesion molecule upregulation, and leukocyte recruitment at the blood-brain barrier. mBio. (2020) 11. doi: 10.1128/mBio.01183-20 PMC7407083 32753493

[152] Nguyen LN Ma D Shui G Wong P Cazenave-Gassiot A Zhang X. Mfsd2a is a transporter for the essential omega-3 fatty acid docosahexaenoic acid. Nature. (2014) 509:503–6. doi: 10.1038/nature13241 24828044

[153] Belaunzarán-Zamudio PF Ortega-Villa AM Mimenza-Alvarado AJ Guerra-De-Blas PDC Aguilar-Navarro SG Sepúlveda-Delgado J. Comparison of the impact of zika and dengue virus infection, and other acute illnesses of unidentified origin on cognitive functions in a prospective cohort in chiapas Mexico. Front Neurol. (2021) 12:631801. doi: 10.3389/fneur.2021.631801 33828518 PMC8019918

[154] Zucker J Neu N Chiriboga CA Hinton VJ Leonardo M Sheikh A. Zika virus-associated cognitive impairment in adolescen. Emerg Infect Dis. (2017) 23:1047–8. doi: 10.3201/eid2306.162029 PMC5443451 28518023

[155] Peeling RW Mabey D Kamb ML Chen XS Radolf JD Benzaken AS . Syphilis . Nat Rev Dis Primers. (2017) 3:17073. doi: 10.1038/nrdp.2017.73 29022569 PMC5809176

[156] Ávila-Nieto C Pedreño-López N Mitjà O Clotet B Blanco J Carrillo J. Syphilis vaccine: challenges, controversies and opportunities. Front Immunol. (2023) 14:1126170. doi: 10.3389/fimmu.2023.1126170 37090699 PMC10118025

[157] Janier M Unemo M Dupin N Tiplica GS Potočnik M Patel R. 2020 european guideline on the management of syphilis. J Eur Acad Dermatol Venereol. (2021) 35:574–88. doi: 10.1111/jdv.16946 33094521

[158] Eickhoff CA Decker CF. Syphilis. Dis Mon. (2016) 62:280–6. doi: 10.1016/j.disamonth.2016.03.012 27091635

[159] Jones-Vanderleest JG. Neurosyphilis, ocular syphilis, and otosyphilis: detection and treatment. Am Fam Physician. (2022) 106:122–3. 35977144

[160] Xiang L Zhang T Zhang B Zhang C Cui W Yue W. Positive syphilis serology contributes to intracranial stenosis in ischemic stroke patients. Brain Behav. (2021a) 11:e01906. doi: 10.1002/brb3.1906 33089668 PMC7821556

[161] Xiang L Zhang T Zhang B Zhang C Hou S Yue W. The associations of increased cerebral small vessel disease with cognitive impairment in neurosyphilis presenting with ischemic stroke. Brain Behav. (2021b) 11:e02187. doi: 10.1002/brb3.2187 33998172 PMC8213652

[162] Ghanem KG. Review: neurosyphilis: A historical perspective and review. CNS Neurosci Ther. (2010) 16:e157–68. doi: 10.1111/j.1755-5949.2010.00183.x PMC6493817 20626434

[163] Duering M Konieczny MJ Tiedt S Baykara E Tuladhar AM Leijsen EV. Serum neurofilament light chain levels are related to small vessel disease burden. J Stroke. (2018) 20:228–38. doi: 10.5853/jos.2017.02565 PMC6007291 29886723

[164] Anad A Barker MK Katanga JA Arfanakis K Bridges LR Esiri MM. Vasculocentric axonal nfh in small vessel disease. J Neuropathol Exp Neurol. (2022) 81:182–92. doi: 10.1093/jnen/nlab134 PMC8922195 35086142

[165] Zhou B Yuan Y Zhang S Guo C Li X Li G. Intestinal flora and disease mutually shape the regional immune system in the intestinal tract. Front Immunol. (2020) 11:575. doi: 10.3389/fimmu.2020.00575 32318067 PMC7147503

[166] Jiang N Xiao Y Yuan K Wang Z. Effect of intestinal microbiota on liver disease and its related future prospection: from the perspective of intestinal barrier damage and microbial metabolites. J Gastroenterol Hepatol. (2023) 38:1056–71. doi: 10.1111/jgh.16129 36662612

[167] Senchukova MA. Microbiota of the gastrointestinal tract: friend or foe? World J Gastroenterol. (2023) 29:19–42. doi: 10.3748/wjg.v29.i1.19 36683718 PMC9850957

[168] Rolhion N Chassaing B. When pathogenic bacteria meet the intestinal microbiota. Philos Trans R Soc Lond B Biol Sci. (2016) 371. doi: 10.1098/rstb.2015.0504 PMC5052746 27672153

[169] Wu H Chen X Zhang S Li J. Gut microbiota, the potential biological medicine for prevention, intervention and drug sensitization to fight diseases. Nutrients. (2022) 14. doi: 10.3390/nu14204220 PMC9610464 36296908

[170] Layunta E Buey B Mesonero JE Latorre E. Crosstalk between intestinal serotonergic system and pattern recognition receptors on the microbiota-gut-brain axis. Front Endocrinol (Lausanne). (2021) 12:748254. doi: 10.3389/fendo.2021.748254 34819919 PMC8607755

[171] Wang HX Wang YP. Gut microbiota-brain axis. Chin Med J (Engl). (2016) 129:2373–80. doi: 10.4103/0366-6999.190667 PMC5040025 27647198

[172] Spielman LJ Gibson DL Klegeris A. Unhealthy gut, unhealthy brain: the role of the intestinal microbiota in neurodegenerative diseases. Neurochem Int. (2018) 120:149–63. doi: 10.1016/j.neuint.2018.08.005 30114473

[173] Peh A O’Donnell JA Broughton BRS Marques FZ. Gut microbiota and their metabolites in stroke: A double-edged sword. Stroke. (2022) 53:1788–801. doi: 10.1161/strokeaha.121.036800 35135325

[174] Hu W Kong X Wang H Li Y Luo Y. Ischemic stroke and intestinal flora: an insight into brain-gut axis. Eur J Med Res. (2022) 27:73. doi: 10.1186/s40001-022-00691-2 35614480 PMC9131669

[175] Nelson JW Phillips SC Ganesh BP Petrosino JF Durgan DJ Bryan RM. The gut microbiome contributes to blood-brain barrier disruption in spontaneously hypertensive stroke prone rats. FASEB J. (2021) 35:e21201. doi: 10.1096/fj.202001117R 33496989 PMC8238036

[176] Lin K Peng F He K Qian Z Mei X Su Z. Research progress on intestinal microbiota regulating cognitive function through the gut-brain axis. Neurol Sci. (2024) 45:3711–21. doi: 10.1007/s10072-024-07525-5 38632176

[177] Hernández-Cabanyero C Vonaesch P. Ectopic colonization by oral bacteria as an emerging theme in health and disease. FEMS Microbiol Rev. (2024) 48. doi: 10.1093/femsre/fuae012 PMC11065354 38650052

[178] Peng X Cheng L You Y Tang C Ren B Li Y. Oral microbiota in human systematic diseases. Int J Sci. (2022) 14:14. doi: 10.1038/s41368-022-00163-7 PMC8891310 35236828

[179] Lamont RJ Koo H Hajishengallis G. The oral microbiota: dynamic communities and host interactions. Nat Rev Microbiol. (2018) 16:745–59. doi: 10.1038/s41579-018-0089-x PMC6278837 30301974

[180] Teles R Wang CY. Mechanisms involved in the association between periodontal diseases and cardiovascular disease. Dis. (2011) 17:450–61. doi: 10.1111/j.1601-0825.2010.01784.x PMC3373016 21223455

[181] Genco RJ Sanz M. Clinical and public health implications of periodontal and systemic diseases: an overview. Periodontol 2000. (2020) 83:7–13. doi: 10.1111/prd.12344 32385880

[182] Baker JL Mark Welch JL Kauffman KM McLean JS He X. The oral microbiome: diversity, biogeography and human health. Nat Rev Microbiol. (2024) 22:89–104. doi: 10.1038/s41579-023-00963-6 37700024 PMC11084736

[183] Zangeneh Z Abdi-Ali A Khamooshian K Alvandi A Abiri R. Bacterial variation in the oral microbiota in multiple sclerosis patients. PloS One. (2021) 16:e0260384. doi: 10.1371/journal.pone.0260384 34847159 PMC8631616

[184] Fleury V Zekeridou A Lazarevic V Gaïa N Giannopoulou C Genton L. Oral dysbiosis and inflammation in parkinson’s disease. J Parkinsons Dis. (2021) 11:619–31. doi: 10.3233/jpd-202459 PMC8150470 33646178

[185] Jungbauer G Stähli A Zhu X Auber Alberi L Sculean A Eick S. Periodontal microorganisms and alzheimer disease - a causative relationship? Periodontol 2000. (2022) 89:59–82. doi: 10.1111/prd.12429 35244967 PMC9314828

[186] Li D Ren T Li H Huang M Chen J He Q. Oral microbiota and porphyromonas gingivalis kgp genotypes altered in parkinson’s disease with mild cognitive impairment. Mol Neurobiol. (2024) 61:8631–9. doi: 10.1007/s12035-024-04119-2 38536604

[187] Taguchi A Miki M Muto A Kubokawa K Migita K Higashi Y. Association between oral health and the risk of lacunar infarction in Japanese adults. Gerontology. (2013) 59:499–506. doi: 10.1159/000353707 23942139

[188] Leira Y López-Dequidt I Arias S Rodríguez-Yáñez M Leira R Sobrino T. Chronic periodontitis is associated with lacunar infarct: A case-control study. Eur J Neurol. (2016) 23:1572–9. doi: 10.1111/ene.13080 27418418

[189] Leira Y Rodríguez-Yáñez M Arias S López-Dequidt I Campos F Sobrino T. Periodontitis is associated with systemic inflammation and vascular endothelial dysfunction in patients with lacunar infarct. J Periodontol. (2019) 90:465–74. doi: 10.1002/jper.18-0560 30417380

[190] Delima AJ Oates T Assuma R Schwartz Z Cochran D Amar S. Soluble antagonists to interleukin-1 (Il-1) and tumor necrosis factor (Tnf) inhibits loss of tissue attachment in experimental periodontitis. J Clin Periodontol. (2001) 28:233–40. doi: 10.1034/j.1600-051x.2001.028003233.x 11284536

[191] Nolde M Alayash Z Reckelkamm SL Kocher T Ehmke B Holtfreter B. Downregulation of interleukin 6 signaling might reduce the risk of periodontitis: A drug target mendelian randomization study. Front Immunol. (2023) 14:1160148. doi: 10.3389/fimmu.2023.1160148 37342352 PMC10277556

[192] Slade GD Offenbacher S Beck JD Heiss G Pankow JS. Acute-phase inflammatory response to periodontal disease in the us population. J Dent Res. (2000) 79:49–57. doi: 10.1177/00220345000790010701 10690660

[193] Mu L Jiang L Chen J Xiao M Wang W Liu P. Serum inflammatory factors and oxidative stress factors are associated with increased risk of frailty and cognitive frailty in patients with cerebral small vessel disease. Front Neurol. (2021) 12:786277. doi: 10.3389/fneur.2021.786277 35069415 PMC8770428

[194] Pussinen PJ Alfthan G Jousilahti P Paju S Tuomilehto J. Systemic exposure to porphyromonas gingivalis predicts incident stroke. Atherosclerosis. (2007) 193:222–8. doi: 10.1016/j.atherosclerosis.2006.06.027 16872615

[195] Wang Y Cai X Li H Jin A Jiang L Chen W. Association of intracranial atherosclerosis with cerebral small vessel disease in a community-based population. Eur J Neurol. (2023) 30:2700–12. doi: 10.1111/ene.15908 37294661

[196] Miyatani F Kuriyama N Watanabe I Nomura R Nakano K Matsui D. Relationship between cnm-positive streptococcus mutans and cerebral microbleeds in humans. Dis. (2015) 21:886–93. doi: 10.1111/odi.12360 26205098

[197] Nakano K Hokamura K Taniguchi N Wada K Kudo C Nomura R. The collagen-binding protein of streptococcus mutans is involved in haemorrhagic stroke. Nat Commun. (2011) 2:485. doi: 10.1038/ncomms1491 21952219 PMC3220351

[198] Shiga Y Hosomi N Nezu T Nishi H Aoki S Nakamori M. Association between periodontal disease due to campylobacter rectus and cerebral microbleeds in acute stroke patients. PloS One. (2020) 15:e0239773. doi: 10.1371/journal.pone.0239773 33031428 PMC7544022

[199] Supriya M Christopher R Prabhakar P Chandra SR. Low vitamin D status is associated with inflammatory response in older patients with cerebral small vessel disease. J Neuroimmunol. (2023) 377:578057. doi: 10.1016/j.jneuroim.2023.578057 36921477

[200] Menéndez SG Manucha W. Vitamin D as a modulator of neuroinflammation: implications for brain health. Curr Pharm Des. (2024) 30:323–32. doi: 10.2174/0113816128281314231219113942 38303529

[201] Tan PH Ji J Hsing CH Tan R Ji RR. Emerging roles of type-I interferons in neuroinflammation, neurological diseases, and long-haul covid. Int J Mol Sci. (2022) 23. doi: 10.3390/ijms232214394 PMC9696119 36430870

[202] Ohm M Hosseini S Lonnemann N He W More T Goldmann O. The potential therapeutic role of itaconate and mesaconate on the detrimental effects of lps-induced neuroinflammation in the brain. J Neuroinflammation. (2024) 21:207. doi: 10.1186/s12974-024-03188-3 39164713 PMC11337794

[203] Zanella I. Neuroinflammation: from molecular basis to therapy. Int J Mol Sci. (2024) 25. doi: 10.3390/ijms25115973 PMC11172940 38892158

[204] McDew-White M Lee E Premadasa LS Alvarez X Okeoma CM Mohan M. Cannabinoids modulate the microbiota-gut-brain axis in hiv/siv infection by reducing neuroinflammation and dysbiosis while concurrently elevating endocannabinoid and indole-3-propionate levels. J Neuroinflammation. (2023) 20:62. doi: 10.1186/s12974-023-02729-6 36890518 PMC9993397

[205] Marim FM Teixeira DC Queiroz-Junior CM Valiate BVS Alves-Filho JC Cunha TM. Inhibition of tryptophan catabolism is associated with neuroprotection during zika virus infection. Front Immunol. (2021) 12:702048. doi: 10.3389/fimmu.2021.702048 34335614 PMC8320694

[206] Chen S Han C Wang X Zhang Q Yang X. Alantolactone improves cognitive impairment in rats with porphyromonas gingivalis infection by inhibiting neuroinflammation, oxidative stress, and reducing Aβ Levels. Brain Res. (2024) 1845:149203. doi: 10.1016/j.brainres.2024.149203 39208968

[207] Bath PM Wardlaw JM. Pharmacological treatment and prevention of cerebral small vessel disease: A review of potential interventions. Int J Stroke. (2015) 10:469–78. doi: 10.1111/ijs.12466 PMC4832291 25727737

